# A Role for Tn*6029* in the Evolution of the Complex Antibiotic Resistance Gene Loci in Genomic Island 3 in Enteroaggregative Hemorrhagic *Escherichia coli* O104:H4

**DOI:** 10.1371/journal.pone.0115781

**Published:** 2015-02-12

**Authors:** Piklu Roy Chowdhury, Ian G. Charles, Steven P. Djordjevic

**Affiliations:** 1 The ithree institute, University of Technology Sydney, PO Box 123 Broadway, Sydney, New South Wales, 2007, Australia; 2 NSW Department of Primary Industries, Elizabeth Macarthur Agricultural Institute, Private Bag 4008, Narellan, New South Wales, 2567, Australia; University Medical Center Utrecht, NETHERLANDS

## Abstract

In enteroaggregative hemorrhagic *Escherichia coli* (EAHEC) O104 the complex antibiotic resistance gene loci (CRL) found in the region of divergence 1 (RD1) within *E. coli* genomic island 3 (GI3) contains *bla*_TEM-1_, *strAB*, *sul2*, *tet(A)*A, and *dfrA7* genes encoding resistance to ampicillin, streptomycin, sulfamethoxazole, tetracycline and trimethoprim respectively. The precise arrangement of antibiotic resistance genes and the role of mobile elements that drove the evolutionary events and created the CRL have not been investigated. We used a combination of bioinformatics and iterative BLASTn searches to determine the micro-evolutionary events that likely led to the formation of the CRL in GI3 using the closed genome sequences of EAHEC O104:H4 strains 2011C-3493 and 2009EL-2050 and high quality draft genomes of EAHEC *E. coli* O104:H4 isolates from sporadic cases not associated with the initial outbreak. Our analyses indicate that the CRL in GI3 evolved from a progenitor structure that contained an In2-derived class 1 integron in a Tn*21*/Tn*1721* hybrid backbone. Within the hybrid backbone, a Tn*6029*-family transposon, identified here as Tn*6029C* abuts the *sul1* gene in the 3´-Conserved Segment (-CS) of a class 1 integron generating a unique molecular signature that has only previously been observed in pASL01a, a small plasmid found in commensal *E. coli* in West Africa. From this common progenitor, independent IS*26*-mediated events created two novel transposons identified here as Tn*6029D* and Tn*6222* in 2011C-3493 and 2009EL-2050 respectively. Analysis of RD1 within GI3 reveals IS26 has played a crucial role in the assembly of regions within the CRL.

## Introduction

The German outbreak of *E*. *coli* O104:H4, and a smaller number of sporadic cases of *E*. *coli* O104:H4 infections that followed a few weeks later in France, resulted in an unusually high incidence of haemolytic uremic syndrome (HUS) (850 cases) (as well as bloody diarrhoea (4320 cases) and 82 deaths (total numbers)) [1]. Isolates from these outbreaks, including 2011C-3493 isolated from a US patient with a history of travel to Germany, express Shiga toxin 2, (*stx*_*2*_) which is typically associated with enterohemorrhagic (EHEC) strains. The outbreak strains are however phylogenetically related to enteroaggregative (EAEC) strains. The hybrid features of O104:H4 indicated that they belong to a new lineage known as the enteroaggregative haemorrhagic *E*. *c* *oli* (EAHEC). Strains isolated from the German outbreak and the ones from the sporadic infections in France form a separate clade (Clade 1) distinct from historical enteroaggregative O104:H4 isolates,- such as strain O104:H4 55989 recovered from Central Africa in 1995 [2–4]. EAHEC O104:H4 has also caused cases of HUS and bloody diarrhea in the Republic of Georgia. Strains isolated in 2009 from the Republic of Georgia (2009EL–2050, 2009EL–2071) are closely related to the Clade 1 strains but cluster distinctly from them and the historical isolates [2].

The 2011 outbreak strains and subsequent isolates recovered from sporadic cases of HUS in France were found to be resistant to multiple antibiotics including third generation cephalosporins (ceftiofur and ceftriaxone), ampicillin, streptomycin, trimethoprim, sulfamethoxazole, tetracycline and sulfisoxazole, severely limiting treatment options [4–7]. Unique to the 2011 outbreak strains was a large IncI1 plasmid encoding the CTX-M-15 cephalosporinase [8]. The plasmid has been sequenced [8] and extensively characterised in EAHEC O104:H4 isolates, but a detailed analysis of the antibiotic resistance genes and their arrangement in Genomic island (GI3) has not been determined.

The acquisition and loss of mobile genetic elements can play an important role in the emergence of new pathogens. The acquisition of virulence genes and antibiotic resistance are considered to be significant events in the emergence of new pathogens because of the potential to impact fitness and transmissibility [9]. Comparative whole genome based analyses of O104:H4 isolates has revealed diversity between EAHEC O104:H4 strains [2,4]. Closed genomes now exist for three EAHEC O104:H4 strains including a representative of the German outbreak strain 2011C-3493 and two strains from the republic of Georgia (2009EL-2050 and 2009EL2071). The closed genome sequences provide contextual information of the precise arrangement of resistance genes and is important for understanding the molecular events that generate complex antibiotic resistance gene loci (CRL)[3,10], [11]. Such closed genomes allowed us to determine the precise arrangement of antibiotic resistance genes within GI3 and the molecular events that created the CRL.

GI3 is a hotspot for genetic events within O104:H4 genomes [2,3] and is unique to Clade 1 O104:H4 strains [3]. It is an important region within O104:H4 genomes because it has previously been demonstrated to be mobile [12,13]. GI3 targets a widely-dispersed genomic hotspot consisting of a target 23 bp sequence and consequently it may be significant in the emergence of future MDR pathogens in the *Enterobacteriaceae*. Moreover, it houses a CRL containing the mercury resistance gene cluster (*mer-*module) and the virulence factor Ag43, implicated in biofilm formation [3]. The CRL also contains genes encoding resistance to ampicillin (*bla*_TEM-1_), streptomycin (*strAB*), sulfisoxazole (*sul1* and *sul2*), tetracycline (*tetA*(A)) and trimethoprim (*dfrA7*). Notably, comparative genome studies of O104:H4 were the first to identify the *bla*_TEM1_-*sul2*-*strA*-*strB* gene cluster in a chromosomal location. Previously, this gene cluster had only been identified on plasmid backbones [14–18]. For example, an IncHI2 plasmid from *Salmonella enterica* serovar Typhimurium from Australia carries a *sul2-strA-strB* gene cluster together with plasmid replication genes *repA* and *repC* adjacent to a module encompassing the *bla*_TEM-1_ gene [15]. On the plasmid, these genes comprise a large part of Tn*6029*, a transposon flanked by direct copies of IS*26* [15]. The molecular events that led to the construction of Tn*6029* have been described and involve the insertion of Tn*1*, Tn*2* or Tn*3* in plasmid RSF1010 followed by the independent insertion of three copies of IS*26* [15]. In 2011 a second variant of the transposon, Tn*6029B* [18], was described within the sequence of an IncH1 plasmid, (pHCM1 [30]) isolated from a *Salmonella enterica* serovar Typhi strain from Vietnam in 1993.

IS*26* is recognised to be an important driver in the evolution of CRL within Gram-negative pathogens [11,19–26]. Tn*6029* and related structures have been identified on multiple antibiotic resistance plasmids [14–18,27,28] suggesting they play an important role in the dissemination of the *bla*_TEM1_-*sul2*-*strA*-*strB* cluster within *E*. *coli* and *Salmonella* populations. Given that the *bla*_TEM1_-*sul2*-*strA*-*strB* gene cluster has now been reported to be chromosomally located on GI3 of EAHEC O104:H4 we were interested in determining whether Tn*6029-*family transposons have played a role in the evolution of the chromosomally located CRL within EAHEC O104:H4 genomes.

Here, we present the key defining features of the CRL within GI3 of the finished genomes of the 2011 German outbreak strain, 2011C-3493 (CP003289.1) and the 2009 Republic of Georgia strain, 2009EL-2050 (CP003297.1). Our analysis indicates that IS*26*-mediated events have played a major role in shaping GI3 in EAHEC O104:H4. We propose an evolutionary model for the CRL seen in EAHEC O104:H4 isolates,- based on the genetic signatures we identified from the closed genomes and iterative BLASTn searches of the microbial genome database.

## Materials and Methods

### Strains and sequences analysed

Regions around Genomic island 3 in the finished genomes of EAHEC strains 2011C-3493 (CP003289.1), 2009EL-2050 (CP003297.1) and 2009EL-2071 (CP003301.1) [2] were analysed in detail to decipher the arrangement of genes within the CRL. Strain 2011C-3493 is from the United States from a patient that had recently returned from Germany during the epidemic and is considered representative of German outbreak strains [2]. Strain 2009EL-2050 was recovered in 2009 from a patient with bloody diarrhoea in the Republic of Georgia and is epidemiologically unrelated to the 2011 outbreak strains [2]. In addition, we have also analysed corresponding regions on previously published plasmids—pASL01a (NC_019091.1) [27], pHCM1 (NC_003384.1) [30] and pAKU_1 (AM412236.1) [32].

### Data mining

Defined sequences spanning regions within RD1 in strains 2011C-3493 and 2009EL-2050 were downloaded from GenBank and aligned using Mauve version 2.3.1 [29] as described below. The automated annotations available through NCBI genome database were manually curated using the NCBI ORF finder and iterative BLASTn and BLASTp searches. Number of nucleotides spanning the priming sites of primers L1 and JL-D2 [19] were used to determine the precise location of Tn*6029*-family transposons within RD1 using AmplifyX version 3.1 software.

### BLASTn analysis of draft O104:H4 genomes

Fifty three draft and complete genomes of *Escherichia coli* O104:H4 isolates (taxid:1038927) available in the GenBank NCBI microbial genome database (on 21^st^ July 2014) were queried with BLASTn using sequences representing different regions within the RD1 structure seen in 2009EL-2050 and 2011C-3493, and proposed to represent a progenitor in this study (see details in results section). The DNA sequence of segments 1 and 2, were derived from the genome sequence of isolate 2011C-3493 while that of fragments 3, 4 and 5 were derived from 2009EL-2050. BLAST alignments that exhibited greater than 99.95% identity over 100% of the query sequence length against *E*. *coli* O104:H4 genomes were deemed significant and analysed in detail here. BLAST hits showing sequence identity against varying lengths of the input sequence spanning Tn*6029* (Fragment 4) were also considered because (1) the region is predicted to be evolving rapidly [14,16,17,27,30] and (2) the region had multiple copies of an insertion element which typically generate scaffold breaks during assembly. A visual representation of locations of the different input query start positions as it appears in S3 Table is detailed in S1 Fig.

## Results

### Genetic features of RD1 that distinguish O104:H4 strains 2009EL-2050 and 2011C-3493

**Fig 1:**
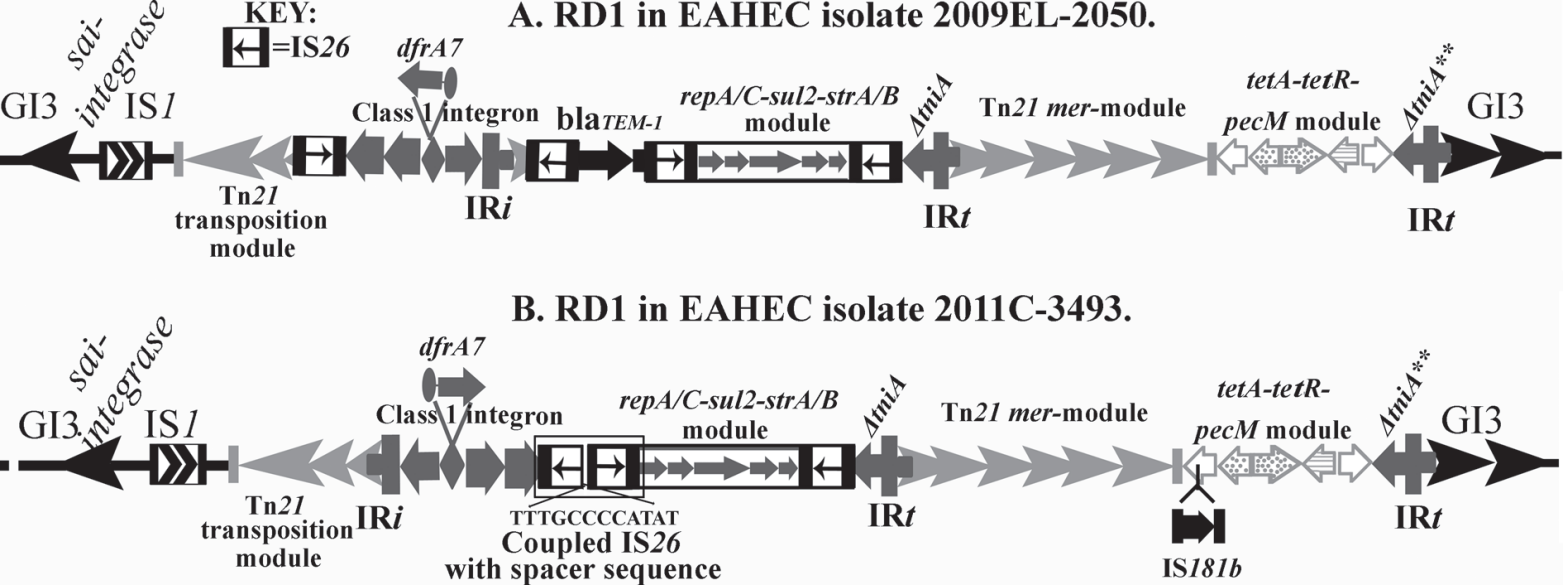
Cartoons defining unique features of the complex resistance loci seen in RD1 of EAHEC O104:H4 isolates. A: RD1 in EAHEC isolate 2009EL-2050. B: RD1 in isolate 2011C-3493. The symbol representing different copies of IS*26* within the resistance loci is highlighted as the key at the top of the figure.

To decipher the salient features of RD1 in 2009EL-2050 (Fig. 1A) and 2011C-3493 (Fig. 1B) we carried out an in-depth sequence analysis and annotation of the regions in both strains. Our analysis showed that the basic backbone of the CRL that defines RD1 in O104:H4 strains comprise a chimeric Tn*21*/Tn*1721* structure with a Tn*1721*-associated *tetA-tetR-pecM* module adjacent to a mercury resistance module from Tn*21*. In both isolates the CRL in RD1 (Fig. 1A and 1B) harbours a class 1 integron, typical of the In2 family, with a *dfrA7* cassette that encodes resistance to trimethoprim [31]. However, the class 1 integron in 2011C_3493 is inverted compared to how it appears in 2009EL-2050 (See Fig. 1A and 1B). The 3´-conserved segment (3´-CS) of the class 1 integron in both strains is partially deleted by the insertion of a composite transposon (see later) and comprises *qacE∆1* and a portion of the *sul1* gene (79 nucleotides at the 3´ end of the *sul1* gene). The deletion in *sul1* and remaining regions of the 3´-CS probably arose by insertion of a composite IS*26* transposon in the 3´-CS followed by an IS*26*-mediated deletion event [19]. These genetic events generated variations in the sequences within RD1 in these strains and will be detailed below.

Downstream of the composite IS*26* transposon within RD1 is the remnant of *∆tniA* missing 428 nucleotides of the gene and the IR*t* inverted repeat, both of which represent key structural components of clinical class 1 integrons (Fig. 1). A mercury resistance module identical to that found in Tn*21* is present beyond IR*t*. The insertion site of the class 1 integron in the mercury module found in EAHEC O104:H4 is typical of the In2-family. The CRL carries a second truncated copy of a *tniA* gene (indicated by *∆tniA* ** in Fig. 1), missing 996 nucleotides from the 3´ end of the gene. *∆tniA* ** (Fig. 1) and its associated IR*t* is found adjacent to the *tetA-tetR-pecM* module, an orientation identical to that seen in Tn*1721*-derived transposons. These data suggests that the two copies of *∆tniA* have separate origins.

**Fig 2:**
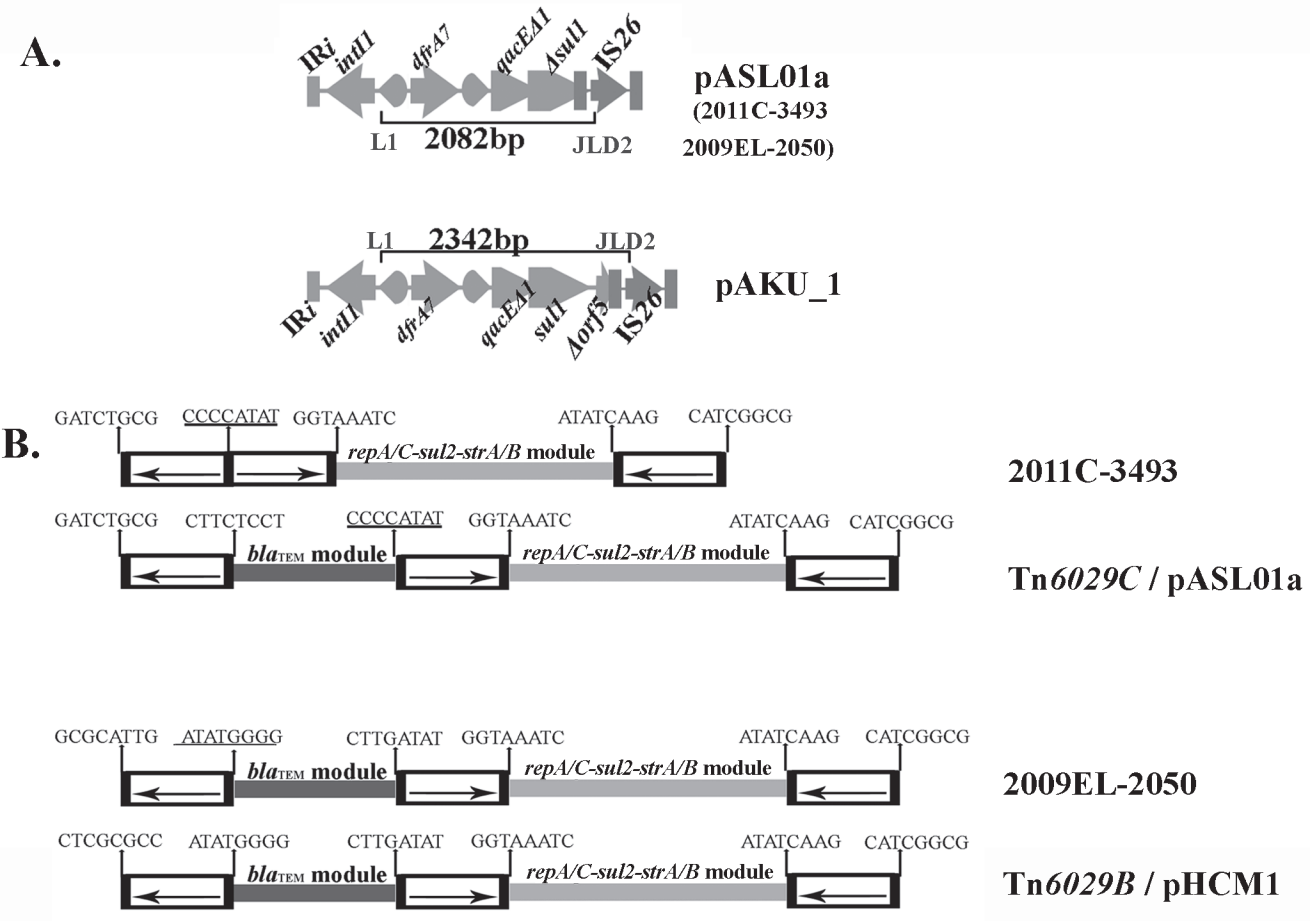
Molecular signatures created by the insertion of IS*26*. A: The 3´-CS of class 1 integrons in pASL01a and pAKU_1 has been structurally modified by different IS*26*-mediated deletion events such that PCR with L1 and JL-D2 primers is expected to generate 2082 and 2342 bp long amplicons respectively. B: Eight base pair signature sequences created by the insertion of IS*26* found flanking the inverted repeats of IS*26* elements clearly suggest that the CRL in strain 2011EC-3493 is a derivative of Tn*6029C* while 2009–2050 is a derivative of Tn*6029B* described previously in pHCM1. The eight base repeat on the left end of Tn*6029B* and that in 2009EL-2050 are different because in pHCM1 (Tn*6029B*) there is a deletion of the class 1 integron whereas in 2009EL-2050 the fragment adjacent to the left hand end of the insertion site is inverted by IS*26*-mediated events. These signatures are also consistent with the proposition that Tn*6029C* evolved from Tn*6029B*.

Notably the 3´-CS of the class 1 integron in both 2011C-3493 and 2009EL-2050 comprises 1210 nucleotides and includes the *qacE∆1* gene plus a partial copy of the *sul1* gene. The *sul1* gene is typically found as a complete ORF in the 3´-CS of most clinical class 1 integrons. An IS*26*-mediated deletion resulted in the loss a region of the 3´-CS leaving only 79 nucleotides at the 3´ end of the *sul1* gene. Previously we have shown that IS*26*-mediated deletion events create novel signatures that can be used in tracking lateral movement of CRL within bacterial populations [16,17,19,20]. *In-silico* PCR simulation using primers in *intI1* (L1) and IS*26* (JL-D2) (see methods) is expected to generate a novel 2082 bp amplicon that can be used as a genetic signature to test for the presence of the CRL in EAHEC O104:H4 (Fig. 2A). Notably, BLASTn analysis of the sequence of the 2082 bp amplicon indicated that it is identical to a homologous fragment in pASL01a (27,072 bp) [27], a small plasmid circulating within commensal *E*. *coli* in West Africa. pASL01a has a backbone of 5,168 bp that carries the plasmid replication genes and Tn*21*-derivative transposon, Tn*ASL01a* [27]. Transposon Tn*ASL01a* encodes resistance to ampicillin, streptomycin, sulfathiazole and trimethoprim, has a class 1 integron carrying *dfrA7* (trimethoprim resistance) gene and an IS*26* transposon which carries the *bla*_TEM1_-*sul2*-*strA*-*strB* gene cluster [27]. The composite IS*26* transposon in pASL01a appears at precisely the same location as in RD1 of 2011C-3493, a remarkable observation that supports the hypothesis that these derivate Tn*21* transposons share evolutionary history.

BLASTn analysis of the 2082 bp amplicon sequence also generated a partial match with plasmid pAKU_1 [32]. Alignments revealed the 2082 fragment is identical across the entire query sequence except for a gap of 260 nt. In pAKU_1, a portion of the *orf5* gene (which is often found associated with 3´-CS of clinical class 1 integrons) is present, which is missing in 2011C-3493 and 2009EL-2050. Detailed sequence similarity searches confirmed that pAKU_1 retains binding sites for primers L1 and JL-D2 and the diagnostic PCR is expected to generate a 2342 bp fragment (Fig. 2) clearly distinguishing the evolutionary histories of the CRL found in pAKU_1 with pASL01a, 2009EL-2050 and 2011C-3493.

### Evidence of Tn*6029*-derived regions within RD1

Within the 3´-CS*∆* and the IR*t* of the class 1 integron in outbreak isolate 2011C-3493 are coupled IS*26* elements (indicated by a box in Fig. 1B) which have their respective transposase genes facing away from each other, followed by *repA-repC-sul2-strA-strB* module and a third copy of IS*26*. Boundaries of the insertion element IS*26* are defined by the presence of 14 nucleotide inverted repeats (TTTGCAACAGTGCC) at either end (IR left or IR_l_ and IR right or IR_r_). Insertion of an IS*26* creates a direct repeat of eight nucleotides at the insertion site. Eight nucleotides long sequences directly abutting IS*26* can therefore be used as a signature to track the evolutionary events that constructed the CRL [19]. Within the coupled IS*26* structure we i) identified a deletion of 10-nucleotides of IS*26*-IR_*l*_ and ii) addition of an eight nucleotides CCCCATAT. As a consequence, a 12-nucleotide spacer signature, TTTGCCCCATAT, separates the two copies of IS*26* in the coupled IS*26* structure (Fig. 1B). Independent BLASTn analysis of the module consisting of the *repA-repC-sul2-strA*-*strB* bounded by two copies of IS*26* indicated 100% identity to the module seen in pASL01a, pHCM1 and other plasmids that carry Tn*6029*-family transposons.

**Fig 3:**
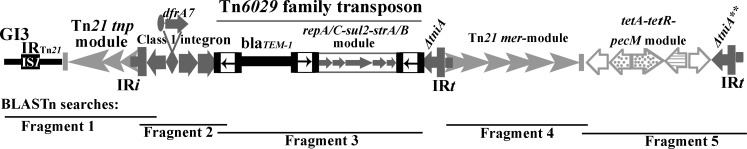
Structure of a proposed progenitor locus from which RD1 has evolved. Lines under the structure represent segments, sequence of which formed the query input sequence in BLASTn analysis of the NCBI Microbial genome database.

In isolate 2009EL-2050, the region flanked by direct copies of IS*26* between IR*i* and IR*t* had two separate modules, one which had *∆tnpR*-*bla*_TEM1_-*∆*CR2 genes, while the other had *repA-repC-sul2-strA*-*strB* genes (Fig. 1A). Independent BLASTn analysis of the entire IS*26* transposon structure containing both the *∆tnpR*-*bla*_TEM1_-*∆*CR2 and *repA-repC-sul2-strA*-*strB* modules showed 99.99% sequence identity to transposon Tn*6029B*, first described in plasmid pHCM1 (Fig. 2B). Based on our analysis of the CRL present in the two genomes the simplest likely progenitor that can give rise independently to the two CRL via separate micro-evolutionary events within a very short time is depicted in Fig. 3. Details of these micro-evolutionary events are explained below.

A comparison of the CRL found in strains 2011C-3943 and 2009EL-2050 showed: i) differences in the number, location and orientation of IS*26* elements, ii) the orientation of the class 1 integron (5´-CS and deleted 3´-CS) in 2009EL-2050 is inverted compared to that of 2011C-3493 and iii) that the module containing *bla*_TEM-1_ gene is absent in the outbreak strain 2011C-3493 but present in progenitor strain 2009EL-2050 (Fig. 1B and 1A respectively).

pASL01a deserves special mention in this context as it carries an identical copy of the class 1 integron containing *dfrA7* with an IS*26*-flanked transposon inserted at an identical location of the 3´-CS as is found in both O104:H4 genomes: 2009EL-2050 and 2011C-3493. Our analysis indicates that Tn*ASL01a* contains a novel variant of Tn*6029*. The Tn*6029* variant is characterized by an 11 bp deletion in the ***∆*** *tnpR*-*bla*_TEM-1-_ΔCR2 segment seen in Tn*6029B* and by the inverted orientation of the module compared to the structures seen in Tn*6029* or Tn*6029B*. We propose the name Tn*6029C* for the Tn*6029* variant present in pASL01a. Characteristic features of Tn*6029* variants and the unique attributes that distinguish Tn*6029B* from Tn*6029C* are shown in S2B Fig.

Tn*6029* and variants Tn*6029B* and Tn*6029C* each carry the *repA*-*repC*-*sul2*-*strA-strB* gene cluster acquired from RSF1010 and *bla*_TEM-1_ from Tn*1*, Tn*2* or Tn*3* [15,33]. The description of the events that led to the formation of Tn*6029* has been reported [15]. Tn*6029B* [18] differs from Tn*6029* by loss of 85 nucleotides in Δ*CR2* gene. Tn*6026C* was likely formed by an IS*26*-mediated inversion of the *bla*_TEM-1_ module followed by deletion of 11 nucleotides within the Tn*2*-derivate (S2B Fig.). This inversion event in Tn*6029C* has also reversed the characteristic eight nucleotide direct repeat signature sequences that abut IS*26* elements in Tn*6029*B (Fig. 2B). Apart from CTCGCGCC in pHCM1, all eight nucleotide signature sequences adjacent to the different copies of IS*26* present in Tn*6029B* and Tn*6029C* were identical to that seen in RD1 in isolates 2009EL-2050 and 2011C-3493 respectively (Fig. 2B).

### Analysis of RD1 in EAHEC O104:H4 genomes

We interrogated the microbial genome database in NCBI for the presence of RD1 in GI3. Our searches were restricted to complete and draft genomes of *E coli* O104:H4 isolates (taxid: 1038927) that aligned to five separate fragments spanning RD1 (See Fig. 3). The rationale for our BLASTn approach resides in our hypothesis that the CRL seen in genomes 2011C-3493 and 2009EL-2050 are the product of independent micro-evolutionary events and therefore we did not want to restrict our searches to the specific structures seen in isolates 2011C-3493 or 2009EL-2050. A summary of the entire BLASTn searches is presented in Table 1, while detailed results of all the BLAST analysis are presented in S1–S5 Tables. On the 20^th^ of July 2014, the GenBank microbial genomic database consisted of 53 *E coli* O104:H4 genomes including the three completely finished genomes described by Ahmed et al., [2] and five well-scaffolded draft genomes with an N50 of 1Mbp [3]. Notably, BLASTn analysis of Fragment 2 (Fig. 3), which includes the 2082 bp signature sequence generated matches with 100% sequence identity in 35 of the 53 O104:H4 genomes. BLASTn searches using Fragment 1 (Fig. 3) which spans a portion of GI3 and its junction with the Tn*21*-specific transposition module and a small overlapping portion with Fragment 2 marking the beginning of integron In2 showed 100% identity in 42 of the 53 genomes. Fragment 4, which spans the Tn*21 mer*-module (Fig. 3), showed 100% identity in 37 genomes. Thirty-six genomes also had the Tn*1721*-derived *tetA-tetR-pecM* module and the associated ΔtniA** gene.

**Table 1:** Summary of the BLASTn analysis of the different fragments considered to contain signature sequences essential for tracing micro-evolutionary events taking place within the CRL of GI3 in *E coli* O104:H4 draft genomes available in GenBank.

Genomes	Tn*21*-*intI1*	*intI1*-IS*26*	Tn*6029*	Tn*21*-mer module	tetA-tetR	O104:H4 Strain
AMWA01000000	+	+	+	+	+	11–03943 (supercontig)
AMVZ010000000		+	+	+	+	11–04080 (supercontig)
AMVY010000000	+	+	+	+	+	11–03439 (supercontig)
AMVX010000000	+	+	+	+	+	11–02913 (supercontig)
AMVW01000000	+	+	+	+	+	11–02318 (supercontig)
AMVV01000000		+	+	+	+	11–02281 (supercontig)
AMVU01000000	+	+	+	+		11–02093 (supercontig)
AMVT01000000	+	+	+	+	+	11–02092 (supercontig)
AMVS01000000	+	+	+	+	+	11–02033–1 (supercontig)
AMVR01000000	+	+	+	+	+	11–02030 (supercontig)
AIPR01000000	+	+	+	+	+	Ec12–0466 [3]
AIPQ01000000	+	+	+	+	+	Ec12–0465 [3]
AHPA01000000	+	+	+	+	+	Ec11–6006
AHOZ01000000	+	+	+	+	+	Ec11–5603
AHOY01000000	+	+	+	+	+	Ec11–4988
AHOX01000000	+	+	+	+	+	Ec11–4987
AHOW01000000	+	+	+	+	+	Ec11–4986
AHOV01000000	+	+	+	+	+	Ec11–5604
AHOU01000000	+	+	+	+	+	Ec11–4984
AGWH01000000	+	+				Ec11–9941[3]
AGWG01000000	+	+				Ec11–9990 [3]
AGWF01000000	+	+	+		+	Ec11–9459[3]
AFWC01000000	+	+			+	H112180283 CS250
AFWB01000000		+				H112180540 CS471
AFVR01000000		+		+		TY2482
AFST01000000	+	+		+	+	C227–11
AFSO01000000	+	+			+	H112180282 CS30
AFPN02000000	+	+			+	H112180280
AFOB02000000	+	+		+		LB226692
NC_019091.1		+	+			pASL01a [27]
NC_018650.1	+	+	+	+	+	2009EL-2050 (Complete [2])
NC_018658.1	+	+	+	+	+	20011C-3493 (Complete [2])
NC_018661.1	+	+	+			2009EL-2071(Complete[2])
AMWA01000000	+	+	+	+	+	11–03943 (supercontig)
AMVZ01000000		+	+	+	+	11–04080 (supercontig)
AMVY01000000	+	+	+	+	+	11–03439 (supercontig)

Fragment 3, spanning the Tn*6029B* transposon, was also used as a query sequence in BLASTn searches. Four genomes, including 2009EL-2050, have Tn*6029B* while 28 genomes appeared to have a substantial segment of the *bla*_TEM-1_ module as indicated by the different start sites of the input query in S3 Table. Our analysis indicates that a variant of Tn*6029* is present in most of the EAHEC O104:H4 genomes. Unpublished genomes of Ec11–4984 (AHOU01000000), Ec11–4988 (AHOY01000000) and Ec11–4986 (AHOW01000000) submitted as supercontigs by the Broad Institute Genome Sequencing Centre for Infectious Diseases appeared to have copies of Tn*6029B* that are identical to those in 2009EL-2050 located within in a single contig.

**Fig 4:**
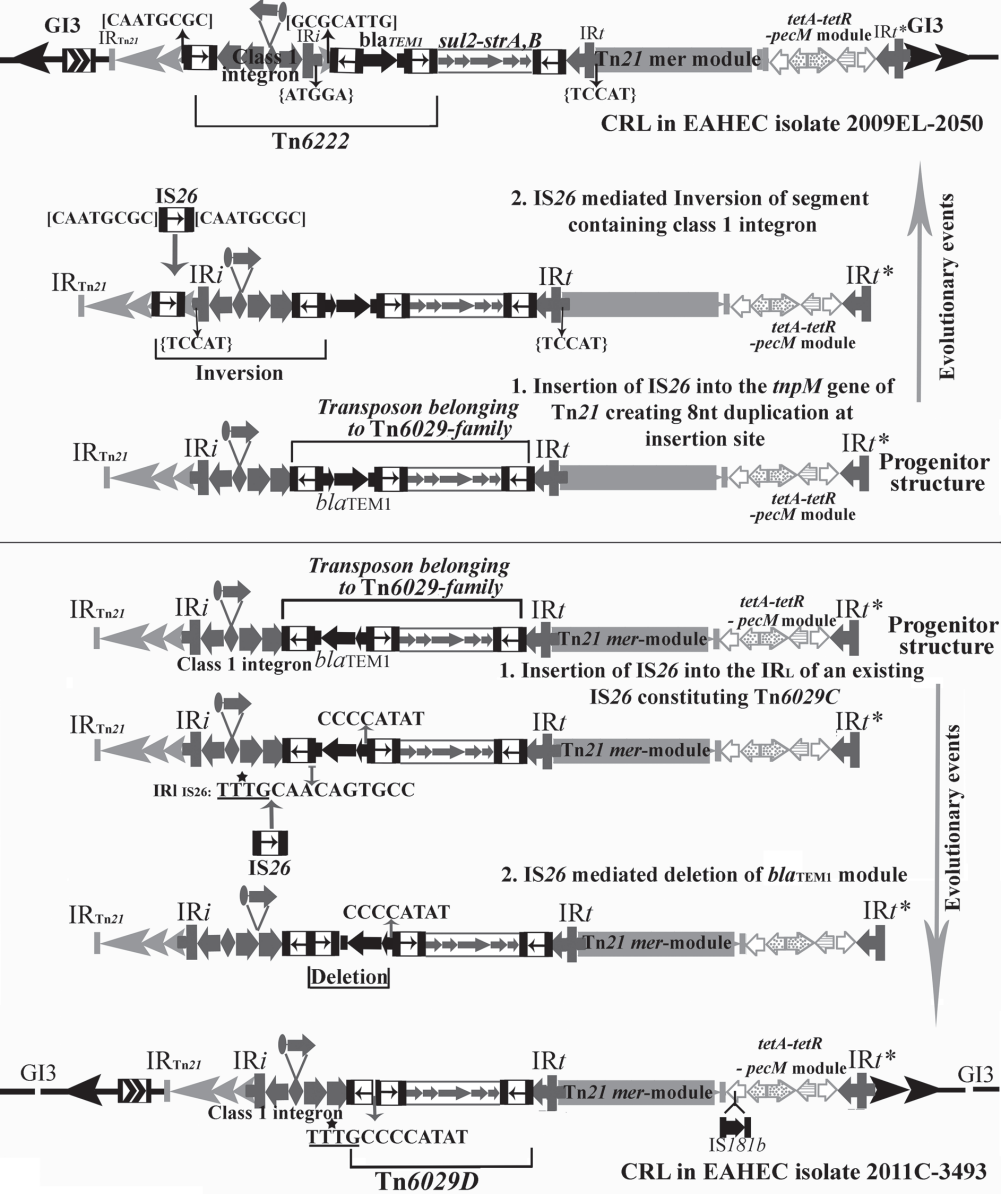
Sequence of events that led to the formation of RD1 in EAHEC O104:H4 isolates 2009EL-2050 from the Republic of Georgia and 2011C-3493, the German HUS representative isolate. A: Events that shaped RD1 in 2009EL-2050. The progenitor was modified by IS*26* inserting into the *tnpM* gene creating a characteristic eight base duplication followed by the inversion of a fragment of DNA bounded by two IS*26* elements and containing a deleted class 1 integron. These events created a new composite IS*26* transposon, Tn*6222*. Direct repeats and reverse complementary sequences generated by IS*26* mediated events are depicted with square brackets [CAATGCGC] and the class 1 integron within curved brackets {TCCAT}. B: Events that shaped RD1 in 2011C-3493. Insertion of IS*26* within the IR_L_ of an existing IS*26* in the progenitor CRL followed by a deletion of *bla*_TEM-1_ gene containing module in Tn*6029*C generated the unique CRL depicted in the outbreak isolate 2011C-3493 and led to the formation of the novel transposon Tn*6223* (bottom panel). The 12 bp sequence within Tn*6223* is a unique signature created by the deletion of the proposed *bla*_TEM-1_ gene containing fragment within Tn*6029C*. The asterisk on the underlined sequences within Tn*6223* is the portion which is identical to IR_L_ of IS*26* and the remaining eight of the 12 bases indicate the eight base region which abuts the IS*26* at the other end.

Table 1 depicts a summary of BLASTn searches using Fragments 1–5. Our data indicates that 22 of the 53 (~42%) O104:H4 genomes in the NCBI microbial genome database have all five fragments that span the progenitor RD1 structure depicted in Fig. 3. Genomes Ec11–4984 (AHOU01000000), Ec11–4988 (AHOY01000000) and Ec11–4986 (AHOW01000000) carry Tn*6029B* in addition to the other four fragments spanning the CRL in our proposed progenitor structure (see Fig. 3). While RD1 in isolate Ec11–4984 (AHOU01000000) is split into two scaffolds, isolates Ec11–4988 and Ec11–4986 have a complete RD1 in one supercontig. Collectively our data supports the presence of the progenitor structure/s depicted in Figs. 3 and 4 in EAHEC O104:H4 isolates from around the world. Isolate Ec12–0466 (AIPR01000000) has a significant portion of the Tn*6029B* and all other segments that constitute the CRL in RD1 in two separate contigs [3]. The contig that has Tn*6029B*, Tn*21* and the Tn*1721* modules starts at a point just past the *bla*_TEM1_ gene of the *∆tnpR*-*bla*_TEM1_-*∆*CR2 module. Similar scenarios were noted in 14 other incomplete genome sequences (S3 Table). Given that assembly of genome sequences using next generation sequencing technologies is problematic, specifically in regions that have multiple copies of any insertion sequences, our search of specific fragments that contain distinguishing features of CRL identified from our analysis of the two completely finished genomes (2011C-3493 and 2009EL-2050) suggests that variants of the proposed progenitor/s are most likely present within GI3 of the majority of O104:H4 genomes.

### The chimeric Tn*21*-Tn*1721* backbone present in the CRL of RD1

The chimeric backbone structure seen in isolates 2011C-3943 and 2009EL-2050 has most likely formed by a homologous recombination event between two In2 derivatives, one of which is identical to that seen in plasmid pASL01a (NC_019091.1) [27] and the other found in *Salmonella enterica* plasmids like pYT2 (AB605179.1) or pSal8934b (JF274992) [34] (see S2A Fig.). The fact that the two copies of *ΔtniA* (*ΔtniA* and ΔtniA** seen in Fig. 1) found in the CRL can be traced to their exact locations in both plasmids supports this contention. pASL01a or variants of it are likely progenitors of the region encompassing the In2 integron because it has the 2082 bp (Fig. 2A) molecular signature seen in 35 of 53 sequenced EAHEC O104:H4 strains. In the outbreak strain 2011C-3943 the *tetA* gene has been interrupted by the insertion of IS*181b* at a time post homologous recombination in the Tn*21*-Tn*1721* backbones, as 10 nucleotide direct repeats of the sequence at the insertion site of IS*181b* and directly abutting the inverted repeats of IS*181b* are clearly evident. The progenitor (Fig. 3) from which RD1 in O104:H4 isolates 2011C-3943 and 2009EL-2050 evolved was likely a hybrid structure that included an In2 derivative as the insertion site of the class 1 integron on the Tn*21* backbone is characteristic of In2 integrons. Our BLASTn analysis of the O104:H4 microbial genomic database using overlapping fragments 4 and 5 (see Fig. 3) which span regions of RD1 containing the Tn*21*-specific and the Tn*1721*-derived modules of CRL (Table 1) provide evidence that these hybrid structures are frequently present in O104:H4 EAHEC.

## Discussion

Multiple comparative genomic studies on both finished and draft genomes of EAHEC O104:H4 [4,35] indicate that RD1 within GI3 is a hotspot for micro-evolutionary events mediated by mobile genetic elements, particularly IS*26* [2]. The preponderance of IS*26* in GI3 indicates that clinically-relevant antibiotic resistance genes known to be mobilised by IS*26* including *bla*_SHV-11,_ *bla*_SHV-12_ (β-lactam resistance) [11,36,37], *qnrB19* (quinolone resistance) [14,36] and *aphA1* (kanamycin/neomycin resistance) [15] can readily recombine within the CRL in O104:H4. Furthermore, homologous recombination events involving IS*26* is well known to drive the transfer of genetic loci between chromosome and plasmid backbones [11,38], and has played a central role in the evolution of new transposon-like structures (This study, [15,40]). As a consequence, evolution of resistance to clinically important antibiotics in O104:H4 should be monitored.

Our analyses indicate that two separate evolutionary scenarios (Fig. 4) shaped the CRL in EAHEC O104:H4 isolates 2011C-3943 and 2009EL-2050. The formation of a chimeric backbone structure formed by a homologous recombination event between two In2 derivatives is a key event in the creation of a Tn*21*/Tn*1721* hybrid transposon that represented a progenitor structure shown in Fig. 3. The CRL seen in 2009EL-2050 and 2011C-3493 evolved separately from Tn*6029B* and Tn*6029C* respectively. The sequence of eight nucleotides adjacent to IS*26* elements in the CRL shown in Fig. 2B provides irrefutable evidence that IS*26* played a key role in the evolution of the CRL seen in EAHEC strains 2011C-3493 and 2009EL-2050. The precise events that created the CRL in 2009EL-2050 is depicted in Fig. 4A and B.

In isolate 2009EL-2050, a copy of IS*26* inserted into the *tnpM* gene of Tn*21*, creating an eight base sequence duplication (Fig. 2B) at the insertion site, in the proposed progenitor. This event created inverted copies of IS*26* flanking the class 1 integron allowing this region to undergo an IS*26*-mediated inversion. Inversion events driven by inverted copies of IS*26* have been previously described [15]. These events generated a novel transposon flanked by direct copies of IS*26* which we have identified here as Tn*6222*. Tn*6222* comprises two modules, one composed of a functional class 1 integron containing a *dfrA7* gene cassette and another with the *bla*_TEM-1_ gene (Fig. 4A). The class 1 integron in transposon Tn*6222* has the ability to assemble more resistance genes via the acquisition of resistance gene cassettes and may continue to evolve from the resistance phenotype imparted by its present configuration.

The CRL in 2011C-3493 has evolved by a separate set of events mediated by the insertion of an IS*26* within the boundaries of Tn*6029C*. IS*26* increasingly plays a key role in the evolutionary events that underpin the formation of CRL in genomic islands among a range of emerging pathogens [21,39]. We propose that a copy of IS*26* inserted into the IR_L_ of the existing IS*26* that abuts the *bla*_TEM-1_ gene in Tn*6029C* in the proposed progenitor (Fig. 4B). A subsequent deletion event removed one copy of IS*26*, the *bla*_TEM-1_ gene and ΔCR2 generating a new derivative of the Tn*6029*-family, identified here as Tn*6029D* (Fig. 4B) This loss of *bla*_TEM1_ in the outbreak strain is consistent with the formation of a translocatable unit [40] formed as a consequence of the insertion of an IS*26* at the IR_L_ of an existing IS26 present in the progenitor structure. While Tn*6029D* is clearly related to Tn*6029C* by virtue of the 12-nucleotide signature sequence (TTTGCCCCATAT), the loss of *bla*_TEM-1_ is unique (Fig. 4B). Evidence that the insertion of IS*26* has driven the creation of the CRL in 2011C-3493 is found in the eight base characteristic sequences shown in Fig. 2B.

The progenitor structure depicted in S2A Fig. was created by a double reciprocal cross-over between the common regions in two In2 derivatives, one of which is identical to plasmid pASL01a (NC_019091.1) [27] and the other identical to that in *Salmonella enterica* plasmids like pYT2 (AB605179.1) or pSal8934b (JF274992) [34]. Although a similar structure is also found in pHCM1, this plasmid is unlikely to be a progenitor of the structure seen in RD1 as it lacks a class 1 integron [15,30]. The insertion of IS*26* generates eight base direct repeats [41] at the site of insertion and is known to induce deletions of variable length creating unique molecular signatures. We acknowledge that one of the limitations in our evolutionary models is that they are based on polished genome sequences of only two EAHEC isolates. To counter this, we performed extensive BLASTn analysis of 53 O104:H4 *E*. *coli* genomes in the microbial genome database. Our analyses confirm the presence of our proposed ancestral structures and identified possible variants of RD1 found in the genomes of 2011C-3493 and 2009EL-2050. In our opinion the major hindrance to rigorous assessment of evolutionary history for complex resistance loci or microbial genomes as a whole is not the quality of available genome data but a lack of statistical models for the inference of genome evolution and the availability of computational tools implementing the models. As genomes evolve their loci undergo rearrangement, insertions, deletions, segmental duplication and lateral transfer. Although efforts to combine these events into a unified statistical model of evolutionary history are ongoing [42,43], no software implementing such a unified inference model is yet available.

The 2082 bp molecular signature has been observed in pASL01a (JQ480155.1), in the CRL of EAHEC O104:H4 German outbreak strain 2011C-3493 (CP003289.1) and strains 2009EL-2050 (CP003297.1) and 2009EL-2071 (CP003301.1) from patients with bloody diarrhoea in the Republic of Georgia, and in a clinical *E*. *coli* isolate (HM999792.1) from Sydney, Australia. In strain 2009EL-2071 the CRL in RD1 has been lost [2]. RD1 is located in GI3 adjacent to the *selC* tRNA gene in EAHEC [3]. GI3 likely presents an evolutionary advantage to strains that carry it because it carries *ag43*, a gene encoding the self-associating serine protease auto-transporter of *Enterobacteriaceae* (SPATE) that influences biofilm formation in EAHEC O104:H4 [44] and the *yeeV*/*yeeU* toxin/antitoxin system [35]. This view is supported by a study showing that kidney damage and virulence gene expression (*stx*_2_, *aggR* and *pgaA*) correlates with the ability of O104:H4 to form biofilms in germ-free mice [45]. GI3 is potentially mobile [12] and targets the 23 bp genomic sequences that are widely dispersed in *E*. *coli* genomes, suggesting it may be a significant player in future emerging pathogens.

## Supporting Information

S1 Fig Diagrammatic guides to interpret the physical locations of the nucleotide alignment start positions (BLASTn analysis of fragment 3) along the sequence corresponding to the Tn*6029*-family transposon seen in isolate 2009EL-2050. Different segments of the transposon have aligned to the different scaffolds of the draft genome sequences in the BLASTn analysis of the fragment 3 (Fig. 3) against draft *E*. *coli* O104:H4 genomes available in GenBank.
(TIF)

S2 Fig A: Homologous recombination events that have shaped the CRL in the theoretical progenitor structure. The homologous recombination event has likely taken place between two multiple antibiotic resistance plasmids pASL01a and plasmids from *Salmonella* like pYT2. The event created a CRL encoding *dfrA7* (resistance to trimethoprim), *bla*_TEM-1_ (resistance to ampicillin), *strAB* (resistance to streptomycin), *sul2* (resistance to sulfamethoxazole), *qacE∆1* (resistance to quaternary compounds), *merA* (resistance to mercury chloride) and *tet(A)*A (resistance to tetracycline). B: A cartoon depicting the structural differences and genetic signatures present in members of Tn*6029*-family of transposons, i.e. Tn*6029*, Tn*6029B* and Tn*6029C*. The top panel shows the structure of Tn*6029* and the middle panel shows that Tn*6029B* is characterised by an 85 bp deletion in the *bla*_TEM_ module (see asterix). In Tn*6029C* (bottom panel), the *bla*_TEM_ containing module is orientated in the reverse direction compared to Tn*6029* / Tn*6029B* and has lost an additional 11 bp from the deleted-CR2 region (indicated by the asterix). Tn*6029C* is the arrangement seen in pASL01a.
(TIF)

S1 Table Results of BLASTn analysis of E coli O104:H4 draft genomes using Fragment 1 (5730nt). (DOCX)

S2 Table Results of BLASTn analysis of E coli O104:H4 draft genomes using Fragment 2 (3298nt). (DOCX)

S3 Table Results of BLASTn analysis of E coli O104:H4 draft genomes using Fragment 3 (8677nt). (DOCX)

S4 Table Results of BLASTn analysis of E coli O104:H4 draft genomes using Fragment4 (4670nt). (DOCX)

S5 Table Results of BLASTn analysis of E coli O104:H4 draft genomes using Fragment 5 (5301nt). (DOCX)

## References

[1] Beutin L, Martin A (2012) Outbreak of Shiga toxin-producing *Escherichia coli* (STEC) O104:H4 infection in Germany causes a paradigm shift with regard to human pathogenicity of STEC strains. J Food Prot 75: 408–418. 10.4315/0362-028X.JFP-11-452 22289607

[2] Ahmed SA, Awosika J, Baldwin C, Bishop-Lilly KA, Biswas B, et al (2012) Genomic comparison of *Escherichia coli* O104:H4 isolates from 2009 and 2011 reveals plasmid, and prophage heterogeneity, including shiga toxin encoding phage stx2. PLoS One 7: e48228 10.1371/journal.pone.0048228 23133618 PMC3486847

[3] Grad YH, Godfrey P, Cerquiera GC, Mariani-Kurkdjian P, Gouali M, et al (2013) Comparative genomics of recent Shiga toxin-producing *Escherichia coli* O104:H4: short-term evolution of an emerging pathogen. MBio 4: e00452–00412. 10.1128/mBio.00452-12 23341549 PMC3551546

[4] Grad YH, Lipsitch M, Feldgarden M, Arachchi HM, Cerqueira GC, et al (2012) Genomic epidemiology of the *Escherichia coli* O104:H4 outbreaks in Europe, 2011. Proc Natl Acad Sci U S A 109: 3065–3070. 10.1073/pnas.1121491109 22315421 PMC3286951

[5] Gault G, Weill FX, Mariani-Kurkdjian P, Jourdan-da Silva N, King L, et al (2011) Outbreak of haemolytic uraemic syndrome and bloody diarrhoea due to *Escherichia coli* O104:H4, south-west France, June 2011. Euro Surveill 16 21749817 10.2807/ese.16.26.19905-en

[6] Jourdan-da Silva N, Watrin M, Weill FX, King LA, Gouali M, et al (2012) Outbreak of haemolytic uraemic syndrome due to Shiga toxin-producing *Escherichia coli* O104:H4 among French tourists returning from Turkey, September 2011. Euro Surveill 17 22297137 10.2807/ese.17.04.20065-en

[7] Mariani-Kurkdjian P, Bingen E, Gault G, Jourdan-Da Silva N, Weill FX (2011) *Escherichia coli* O104:H4 south-west France, June 2011. Lancet Infect Dis 11: 732–733. 10.1016/S1473-3099(11)70266-3 21958580

[8] Kunne C, Billion A, Mshana SE, Schmiedel J, Domann E, et al (2012) Complete sequences of plasmids from the hemolytic-uremic syndrome-associated *Escherichia coli* strain HUSEC41. J Bacteriol 194: 532–533. 10.1128/JB.06368-11 22207742 PMC3256666

[9] Riley LW (2014) Pandemic lineages of extraintestinal pathogenic *Escherichia coli* . Clin Microbiol Infect 20: 380–390. 10.1111/1469-0691.12646 24766445

[10] Baquero F, Tobes R (2013) Bloody coli: a gene cocktail in *Escherichia coli* O104:H4. MBio 4: e00066–00013. 10.1128/mBio.00066-13 23422408 PMC3624515

[11] Hudson CM, Bent ZW, Meagher RJ, Williams KP (2014) Resistance determinants and mobile genetic elements of an NDM-1-encoding *Klebsiella pneumoniae* strain. PLoS One 9: e99209 10.1371/journal.pone.0099209 24905728 PMC4048246

[12] Chouikha I, Germon P, Bree A, Gilot P, Moulin-Schouleur M, et al (2006) A *selC*-associated genomic island of the extraintestinal avian pathogenic *Escherichia coli* strain BEN2908 is involved in carbohydrate uptake and virulence. J Bacteriol 188: 977–987. 16428402 10.1128/JB.188.3.977-987.2006 PMC1347334

[13] Blum G, Ott M, Lischewski A, Ritter A, Imrich H, et al (1994) Excision of large DNA regions termed pathogenicity islands from tRNA-specific loci in the chromosome of an *Escherichia coli* wild-type pathogen. Infect Immun 62: 606–614. 7507897 10.1128/iai.62.2.606-614.1994 PMC186147

[14] Cain AK, Hall RM (2012) Evolution of a multiple antibiotic resistance region in IncHI1 plasmids: reshaping resistance regions in situ. J Antimicrob Chemother 67: 2848–2853. 10.1093/jac/dks317 22888274

[15] Cain AK, Liu X, Djordjevic SP, Hall RM (2010) Transposons related to Tn*1696* in IncHI2 plasmids in multiply antibiotic resistant *Salmonella enterica* serovar Typhimurium from Australian animals. Microb Drug Resist 16: 197–202. 10.1089/mdr.2010.0042 20701539

[16] Venturini C, Beatson SA, Djordjevic SP, Walker MJ (2010) Multiple antibiotic resistance gene recruitment onto the enterohemorrhagic *Escherichia coli* virulence plasmid. FASEB J 24: 1160–1166. 10.1096/fj.09-144972 19917674

[17] Venturini C, Hassan KA, Roy Chowdhury P, Paulsen IT, Walker MJ, et al (2013) Sequences of Two Related Multiple Antibiotic Resistance Virulence Plasmids Sharing a Unique IS*26*-Related Molecular Signature Isolated from Different *Escherichia coli* Pathotypes from Different Hosts. PLoS One 8: e78862 10.1371/journal.pone.0078862 24223859 PMC3817090

[18] Bailey JK, Pinyon JL, Anantham S, Hall RM (2011) Distribution of the *bla* _TEM_ gene and *bla* _TEM_-containing transposons in commensal *Escherichia coli* . J Antimicrob Chemother 66: 745–751. 10.1093/jac/dkq529 21393132

[19] Dawes FE, Kuzevski A, Bettelheim KA, Hornitzky MA, Djordjevic SP, et al (2010) Distribution of class 1 integrons with IS*26*-mediated deletions in their 3'-conserved segments in *Escherichia coli* of human and animal origin. PLoS One 5: e12754 10.1371/journal.pone.0012754 20856797 PMC2939871

[20] Djordjevic SP, Stokes HW, Roy Chowdhury P (2013) Mobile elements, zoonotic pathogens and commensal bacteria: conduits for the delivery of resistance genes into humans, production animals and soil microbiota. Front Microbiol 4: 86 10.3389/fmicb.2013.00086 23641238 PMC3639385

[21] Doublet B, Praud K, Weill FX, Cloeckaert A (2009) Association of IS*26*-composite transposons and complex In4-type integrons generates novel multidrug resistance loci in *Salmonella* genomic island 1. J Antimicrob Chemother 63: 282–289. 10.1093/jac/dkn500 19074421

[22] Lee KY, Hopkins JD, Syvanen M (1990) Direct involvement of IS*26* in an antibiotic resistance operon. J Bacteriol 172: 3229–3236. 2160941 10.1128/jb.172.6.3229-3236.1990 PMC209129

[23] Naas T, Mikami Y, Imai T, Poirel L, Nordmann P (2001) Characterization of In53, a class 1 plasmid- and composite transposon-located integron of *Escherichia coli* which carries an unusual array of gene cassettes. J Bacteriol 183: 235–249. 11114922 10.1128/JB.183.1.235-249.2001 PMC94871

[24] Post V, White PA, Hall RM (2010) Evolution of AbaR-type genomic resistance islands in multiply antibiotic-resistant *Acinetobacter baumannii* . J Antimicrob Chemother 65: 1162–1170. 10.1093/jac/dkq095 20375036

[25] Roy Chowdhury P, Ingold A, Vanegas N, Martinez E, Merlino J, et al (2011) Dissemination of multiple drug resistance genes by class 1 integrons in *Klebsiella pneumoniae* isolates from four countries: a comparative study. Antimicrob Agents Chemother 55: 3140–3149. 10.1128/AAC.01529-10 21518841 PMC3122386

[26] Ruiz E, Rojo-Bezares B, Saenz Y, Olarte I, Esteban I, et al (2010) Outbreak caused by a multi-resistant *Klebsiella pneumoniae* strain of new sequence type ST341 carrying new genetic environments of aac(6')-Ib-cr and qnrS1 genes in a neonatal intensive care unit in Spain. Int J Med Microbiol 300: 464–469. 10.1016/j.ijmm.2010.04.014 20547103

[27] Labar AS, Millman JS, Ruebush E, Opintan JA, Bishar RA, et al (2012) Regional dissemination of a trimethoprim-resistance gene cassette via a successful transposable element. PLoS One 7: e38142 10.1371/journal.pone.0038142 22666464 PMC3364232

[28] Szczepanowski R, Braun S, Riedel V, Schneiker S, Krahn I, et al (2005) The 120 592 bp IncF plasmid pRSB107 isolated from a sewage-treatment plant encodes nine different antibiotic-resistance determinants, two iron-acquisition systems and other putative virulence-associated functions. Microbiology 151: 1095–1111. 15817778 10.1099/mic.0.27773-0

[29] Darling AC, Mau B, Blattner FR, Perna NT (2004) Mauve: multiple alignment of conserved genomic sequence with rearrangements. Genome Res 14: 1394–1403. 15231754 10.1101/gr.2289704 PMC442156

[30] Wain J, Diem Nga LT, Kidgell C, James K, Fortune S, et al (2003) Molecular analysis of incHI1 antimicrobial resistance plasmids from *Salmonella* serovar Typhi strains associated with typhoid fever. Antimicrob Agents Chemother 47: 2732–2739. 12936967 10.1128/AAC.47.9.2732-2739.2003 PMC182646

[31] Partridge SR, Brown HJ, Stokes HW, Hall RM (2001) Transposons Tn*1696* and Tn*21* and their integrons In4 and In2 have independent origins. Antimicrob Agents Chemother 45: 1263–1270. 11257044 10.1128/AAC.45.4.1263-1270.2001 PMC90453

[32] Holt KE, Thomson NR, Wain J, Phan MD, Nair S, et al (2007) Multidrug-resistant *Salmonella enterica* serovar paratyphi A harbors IncHI1 plasmids similar to those found in serovar typhi. J Bacteriol 189: 4257–4264. 17384186 10.1128/JB.00232-07 PMC1913411

[33] Yau S, Liu X, Djordjevic SP, Hall RM (2010) RSF1010-like plasmids in Australian *Salmonella enterica* serovar Typhimurium and origin of their *sul2-strA-strB* antibiotic resistance gene cluster. Microb Drug Resist 16: 249–252. 10.1089/mdr.2010.0033 20617928

[34] Tamamura Y, Tanaka K, Akiba M, Kanno T, Hatama S, et al (2013) Complete Nucleotide Sequences of Virulence-Resistance Plasmids Carried by Emerging Multidrug-Resistant *Salmonella enterica* Serovar Typhimurium Isolated from Cattle in Hokkaido, Japan. PLoS One 8: e77644 10.1371/journal.pone.0077644 24155970 PMC3796477

[35] Guy L, Jernberg C, Arven Norling J, Ivarsson S, Hedenstrom I, et al (2013) Adaptive Mutations and Replacements of Virulence Traits in the *Escherichia coli* O104:H4 Outbreak Population. PLoS One 8: e63027 10.1371/journal.pone.0063027 23675451 PMC3651199

[36] Dionisi AM, Lucarelli C, Owczarek S, Luzzi I, Villa L (2009) Characterization of the plasmid-borne quinolone resistance gene *qnrB19* in *Salmonella enterica* serovar Typhimurium. Antimicrob Agents Chemother 53: 4019–4021. 10.1128/AAC.00294-09 19528272 PMC2737868

[37] Ford PJ, Avison MB (2004) Evolutionary mapping of the SHV beta-lactamase and evidence for two separate IS*26*-dependent *bla* _SHV_ mobilization events from the *Klebsiella pneumoniae* chromosome. J Antimicrob Chemother 54: 69–75. 15163647 10.1093/jac/dkh251

[38] Osborn AM, Bruce KD, Strike P, Ritchie DA (1997) Distribution, diversity and evolution of the bacterial mercury resistance (mer) operon. FEMS Microbiol Rev 19: 239–262. 9167257 10.1111/j.1574-6976.1997.tb00300.x

[39] Siebor E, Neuwirth C (2013) Emergence of Salmonella genomic island 1 (SGI1) among *Proteus mirabilis* clinical isolates in Dijon, France. J Antimicrob Chemother 68: 1750–1756. 10.1093/jac/dkt100 23580563

[40] Harmer CJ, Moran RA, Hall RM (2014) Movement of IS*26*-Associated Antibiotic Resistance Genes Occurs via a Translocatable Unit That Includes a Single IS*26* and Preferentially Inserts Adjacent to Another IS*26* . MBio 5 10.1128/mBio.02283-14 25293759 PMC4196232

[41] Mollet B, Iida S, Arber W (1985) Gene organization and target specificity of the prokaryotic mobile genetic element IS26. Mol Gen Genet 201: 198–203. 3003524 10.1007/BF00425660

[42] Boussau B, Szollosi GJ, Duret L, Gouy M, Tannier E, et al (2013) Genome-scale coestimation of species and gene trees. Genome Res 23: 323–330. 10.1101/gr.141978.112 23132911 PMC3561873

[43] Sjostrand J, Tofigh A, Daubin V, Arvestad L, Sennblad B, et al (2014) A Bayesian method for analyzing lateral gene transfer. Syst Biol 63: 409–420. 10.1093/sysbio/syu007 24562812

[44] Vejborg RM, Klemm P (2009) Cellular chain formation in *Escherichia coli* biofilms. Microbiology 155: 1407–1417. 10.1099/mic.0.026419-0 19383712

[45] Al Safadi R, Abu-Ali GS, Sloup RE, Rudrik JT, Waters CM, et al (2012) Correlation between in vivo biofilm formation and virulence gene expression in *Escherichia coli* O104:H4. PLoS One 7: e41628 10.1371/journal.pone.0041628 22848550 PMC3405000

