# Correction: A Role for Tn*6029* in the Evolution of the Complex Antibiotic Resistance Gene Loci in Genomic Island 3 in Enteroaggregative Hemorrhagic *Escherichia coli* O104:H4

**DOI:** 10.1371/journal.pone.0126197

**Published:** 2015-04-17

**Authors:** The PLOS ONE Staff

The Acknowledgments section is incomplete. The correct Acknowledgments should appear as follows: We thank Aaron Darling and Hatch Stokes for their comments on an early draft of this manuscript.

The image for Fig 2 is incorrect. The publisher apologizes for the error. Please see the corrected Fig 2 here.

**Fig 2:**
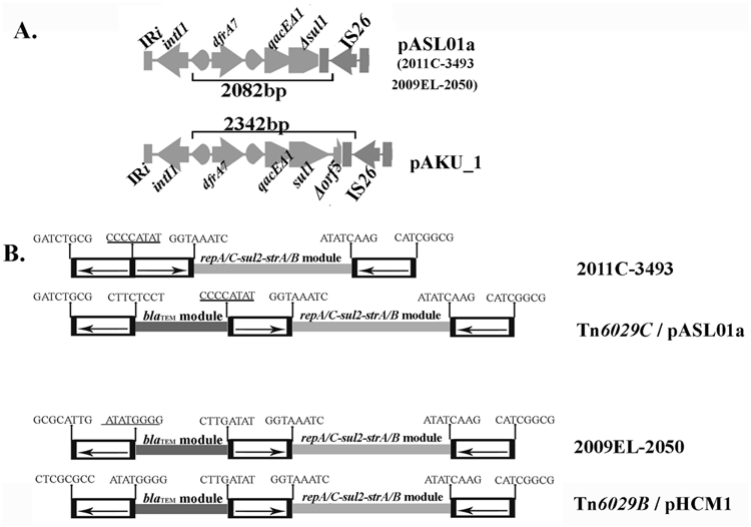
Molecular signatures created by the insertion of IS*26*. A: The 3´-CS of class 1 integrons in pASL01a and pAKU_1 has been structurally modified by different IS*26*-mediated deletion events such that PCR with L1 and JL-D2 primers is expected to generate 2082 and 2342 bp long amplicons respectively. B: Eight base pair signature sequences created by the insertion of IS*26* found flanking the inverted repeats of IS*26* elements clearly suggest that the CRL in strain 2011EC-3493 is a derivative of Tn*6029C* while 2009–2050 is a derivative of Tn*6029B* described previously in pHCM1. The eight base repeat on the left end of Tn*6029B* and that in 2009EL-2050 are different because in pHCM1 (Tn*6029B*) there is a deletion of the class 1 integron whereas in 2009EL-2050 the fragment adjacent to the left hand end of the insertion site is inverted by IS*26*-mediated events. These signatures are also consistent with the proposition that Tn*6029C* evolved from Tn*6029B*.
