# Supplementary figures and images for: A Role for Tn6029 in the Evolution of the Complex Antibiotic Resistance Gene Loci in Genomic Island 3 in Enteroaggregative Hemorrhagic Escherichia coli O104:H4
Source: PLoS One. 2015 Feb 12;10(2):e0115781. doi: 10.1371/journal.pone.0115781 (PMC4326458; doi:10.1371/journal.pone.0115781)

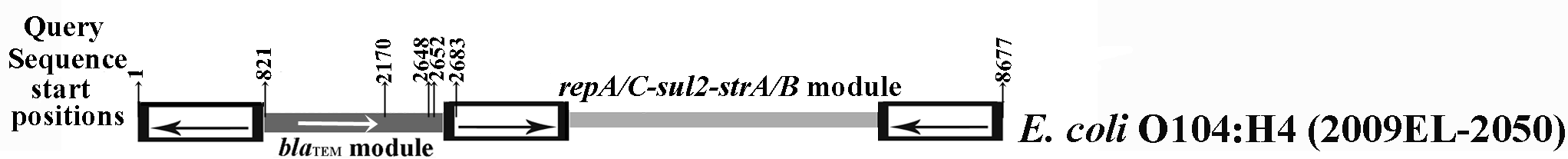

Supplement: S1 Fig — Different segments of the transposon have aligned to the different scaffolds of the draft genome sequences in the BLASTn analysis of the fragment 3 (Fig. 3) against draft E. coli O104:H4 genomes available in GenBank. (TIF) [file pone.0115781.s001.tif]

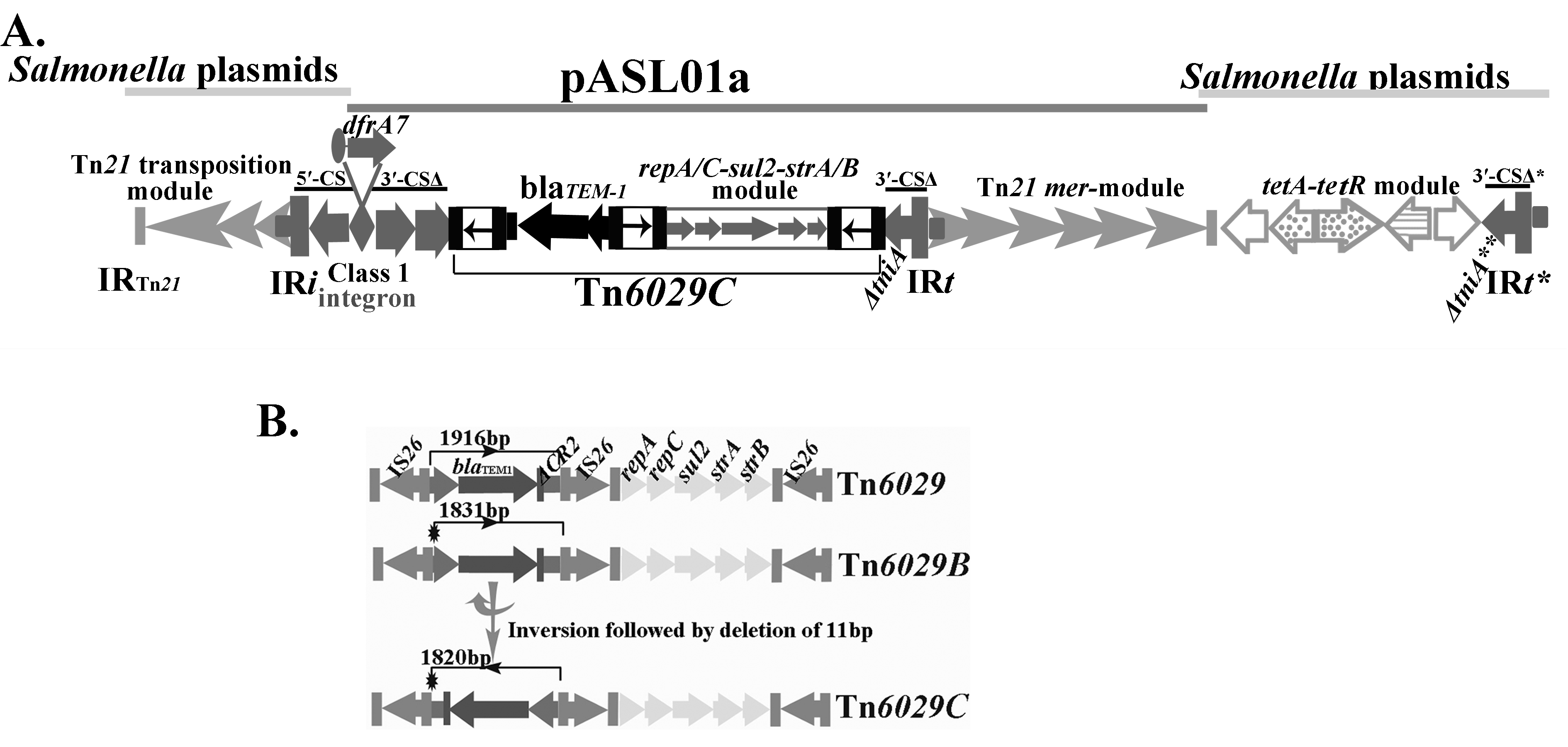

Supplement: S2 Fig — The homologous recombination event has likely taken place between two multiple antibiotic resistance plasmids pASL01a and plasmids from Salmonella like pYT2. The event created a CRL encoding dfrA7 (resistance to trimethoprim), bla TEM-1 (resistance to ampicillin), strAB (resistance to streptomycin), sul2 (resistance to sulfamethoxazole), qacE∆1 (resistance to quaternary compounds), merA (resistance to mercury chloride) and tet(A)A (resistance to tetracycline). B: A cartoon depicting the structural differences and genetic signatures present in members of Tn6029-family of transposons, i.e. Tn6029, Tn6029B and Tn6029C. The top panel shows the structure of Tn6029 and the middle panel shows that Tn6029B is characterised by an 85 bp deletion in the bla TEM module (see asterix). In Tn6029C (bottom panel), the bla TEM containing module is orientated in the reverse direction compared to Tn6029 / Tn6029B and has lost an additional 11 bp from the deleted-CR2 region (indicated by the asterix). Tn6029C is the arrangement seen in pASL01a. (TIF) [file pone.0115781.s002.tif]
