# Supplementary material for: A Role for Tn6029 in the Evolution of the Complex Antibiotic Resistance Gene Loci in Genomic Island 3 in Enteroaggregative Hemorrhagic Escherichia coli O104:H4
Source: PLoS One. 2015 Feb 12;10(2):e0115781. doi: 10.1371/journal.pone.0115781 (PMC4326458; doi:10.1371/journal.pone.0115781)
Supplement: S1 Table — (DOCX) [file pone.0115781.s003.docx]

**Table S1: Results of BLASTn analysis using Fragment 1 (5730nt)**

| **Subject ID** | **% identity** | **Alignment length** | **Mismatches** | **Gaps in align** | **Query start** | **Query end** | **Subject Start** | **Subject End** | **E-Value** | **Bit Score** | **Genomes** |
| --- | --- | --- | --- | --- | --- | --- | --- | --- | --- | --- | --- |
|  |  |  |  |  |  |  |  |  |  |  |  |
| NC_018658.1 | 100 | 5730 | 0 | 0 | 1 | 5730 | 92700 | 86971 | 0 | 10582 | 20011C-3493 |
| AMVY01000007.1 | 99.98 | 5730 | 1 | 0 | 1 | 5730 | 78954 | 84683 | 0 | 10576 |  |
| AMVX01000004.1 | 99.98 | 5730 | 1 | 0 | 1 | 5730 | 826547 | 832276 | 0 | 10576 |  |
| AMVW01000016.1 | 99.98 | 5730 | 1 | 0 | 1 | 5730 | 79062 | 84791 | 0 | 10576 |  |
| AMVU01000003.1 | 99.98 | 5730 | 1 | 0 | 1 | 5730 | 78954 | 84683 | 0 | 10576 |  |
| AMVS01000013.1 | 99.98 | 5730 | 1 | 0 | 1 | 5730 | 587327 | 593056 | 0 | 10576 |  |
| AIPR01000022.1 | 99.98 | 5730 | 1 | 0 | 1 | 5730 | 435350 | 441079 | 0 | 10576 | Ec12-0466 |
| AHPA01000012.1 | 99.98 | 5730 | 1 | 0 | 1 | 5730 | 75336 | 81065 | 0 | 10576 |  |
| AHOZ01000018.1 | 99.98 | 5730 | 1 | 0 | 1 | 5730 | 75432 | 81161 | 0 | 10576 |  |
| AHOY01000021.1 | 99.98 | 5730 | 1 | 0 | 1 | 5730 | 435240 | 440969 | 0 | 10576 |  |
| AHOX01000013.1 | 99.98 | 5730 | 1 | 0 | 1 | 5730 | 75432 | 81161 | 0 | 10576 |  |
| AHOW01000021.1 | 99.98 | 5730 | 1 | 0 | 1 | 5730 | 75547 | 81276 | 0 | 10576 |  |
| AHOV01000018.1 | 99.98 | 5730 | 1 | 0 | 1 | 5730 | 147057 | 152786 | 0 | 10576 |  |
| AGWH01000013.1 | 99.98 | 5730 | 1 | 0 | 1 | 5730 | 75972 | 81701 | 0 | 10576 | Ec11-9941 |
| AFVD01000031.1 | 99.98 | 5730 | 1 | 0 | 1 | 5730 | 75577 | 81306 | 0 | 10576 |  |
| AFVC01000020.1 | 99.98 | 5730 | 1 | 0 | 1 | 5730 | 75405 | 81134 | 0 | 10576 |  |
| AFUX01000021.1 | 99.98 | 5730 | 1 | 0 | 1 | 5730 | 73111 | 78840 | 0 | 10576 |  |
| AFST01000007.2 | 99.98 | 5730 | 1 | 0 | 1 | 5730 | 150239 | 155968 | 0 | 10576 |  |
| AFSO01000030.1 | 99.98 | 5730 | 1 | 0 | 1 | 5730 | 76657 | 82386 | 0 | 10576 |  |
| AFRM01000015.1 | 99.98 | 5730 | 1 | 0 | 1 | 5730 | 436418 | 442147 | 0 | 10576 |  |
| AFPN02000020.1 | 99.98 | 5730 | 1 | 0 | 1 | 5730 | 145593 | 151322 | 0 | 10576 |  |
| AFWC01000250.1 | 99.93 | 5732 | 2 | 2 | 1 | 5730 | 75786 | 81517 | 0 | 10562 |  |
| AFRH01000011.1 | 99.95 | 5731 | 2 | 1 | 1 | 5730 | 75432 | 81162 | 0 | 10565 |  |
| AMWA01000006.1 | 99.97 | 5730 | 2 | 0 | 1 | 5730 | 145298 | 151027 | 0 | 10571 |  |
| AMVT01000002.1 | 99.97 | 5730 | 2 | 0 | 1 | 5730 | 78966 | 84695 | 0 | 10571 |  |
| AMVR01000009.1 | 99.97 | 5730 | 2 | 0 | 1 | 5730 | 78954 | 84683 | 0 | 10571 |  |
| AHOU01000017.1 | 99.97 | 5730 | 2 | 0 | 1 | 5730 | 75596 | 81325 | 0 | 10571 |  |
| AFVA01000009.1 | 99.93 | 5730 | 4 | 0 | 1 | 5730 | 75547 | 81276 | 0 | 10560 |  |
| AFVE01000008.1 | 99.74 | 5730 | 4 | 1 | 1 | 5730 | 75432 | 81150 | 0 | 10488 |  |
| AGWG01000027.1 | 99.9 | 5730 | 6 | 0 | 1 | 5730 | 75432 | 81161 | 0 | 10549 | Ec11-9990 |
| AFRI01000009.1 | 99.9 | 5730 | 6 | 0 | 1 | 5730 | 434748 | 440477 | 0 | 10549 |  |
| AIPQ01000028.1 | 99.98 | 4826 | 1 | 0 | 1 | 4826 | 145213 | 150038 | 0 | 8907 | Ec12-0465 |
| AGWF01000028.1 | 99.98 | 4826 | 1 | 0 | 1 | 4826 | 144952 | 149777 | 0 | 8907 | Ec11-9459 |
| NC_018650.1 | 99.98 | 4826 | 1 | 0 | 1 | 4826 | 94777 | 89952 | 0 | 8907 | 2009EL-2050 |
| NC_018661.1 | 99.98 | 4826 | 1 | 0 | 1 | 4826 | 85206 | 80381 | 0 | 8907 | 2009EL-2071 |
| AFOG01000021.1* | 100 | 4605 | 0 | 0 | 1126 | 5730 | 1 | 4605 | 0 | 8504 | TY-2482 |
| JHLJ01000058.1* | 100 | 4575 | 0 | 0 | 1156 | 5730 | 7356 | 2782 | 0 | 8449 |  |
| AFOB020000300.1* | 99 | 4576 | 1 | 0 | 1155 | 5730 | 8804 | 4229 | 0 | 8421 |  |
| NC_018658.1 | 100 | 5730 | 0 | 0 | 1 | 5730 | 92700 | 86971 | 0 | 10582 | 20011C-3493 |
| AMVY01000007.1 | 99.98 | 5730 | 1 | 0 | 1 | 5730 | 78954 | 84683 | 0 | 10576 |  |
| AMVX01000004.1 | 99.98 | 5730 | 1 | 0 | 1 | 5730 | 826547 | 832276 | 0 | 10576 |  |
| AMVW01000016.1 | 99.98 | 5730 | 1 | 0 | 1 | 5730 | 79062 | 84791 | 0 | 10576 |  |
