# Supplementary material for: A Role for Tn6029 in the Evolution of the Complex Antibiotic Resistance Gene Loci in Genomic Island 3 in Enteroaggregative Hemorrhagic Escherichia coli O104:H4
Source: PLoS One. 2015 Feb 12;10(2):e0115781. doi: 10.1371/journal.pone.0115781 (PMC4326458; doi:10.1371/journal.pone.0115781)
Supplement: S2 Table — (DOCX) [file pone.0115781.s004.docx]

**Table 2: Results of BLASTn analysis using Fragment 2 (3298nt)**

| **Subject ID** | **%identity** | **Alignment length** | **Mismatches** | **Gaps in align** | **Query start** | **Query end** | **Subject Start** | **Subject End** | **E-Value** | **Bit Score** | **Genomes** |
| --- | --- | --- | --- | --- | --- | --- | --- | --- | --- | --- | --- |
|  |  |  |  |  |  |  |  |  |  |  |  |
| AMWA01000006.1 | 100 | 3298 | 0 | 0 | 1 | 3298 | 150611 | 153908 | 0 | 6091 |  |
| AMVZ01000009.1 | 100 | 3298 | 0 | 0 | 1 | 3298 | 4363 | 7660 | 0 | 6091 |  |
| AMVY01000007.1 | 100 | 3298 | 0 | 0 | 1 | 3298 | 84267 | 87564 | 0 | 6091 |  |
| AMVX01000004.1 | 100 | 3298 | 0 | 0 | 1 | 3298 | 831860 | 835157 | 0 | 6091 |  |
| AMVW01000016.1 | 100 | 3298 | 0 | 0 | 1 | 3298 | 84375 | 87672 | 0 | 6091 |  |
| AMVV01000010.1 | 100 | 3298 | 0 | 0 | 1 | 3298 | 4498 | 7795 | 0 | 6091 |  |
| AMVU01000003.1 | 100 | 3298 | 0 | 0 | 1 | 3298 | 84267 | 87564 | 0 | 6091 |  |
| AMVT01000002.1 | 100 | 3298 | 0 | 0 | 1 | 3298 | 84279 | 87576 | 0 | 6091 |  |
| AMVS01000013.1 | 100 | 3298 | 0 | 0 | 1 | 3298 | 592640 | 595937 | 0 | 6091 |  |
| AMVR01000009.1 | 100 | 3298 | 0 | 0 | 1 | 3298 | 84267 | 87564 | 0 | 6091 |  |
| AIPR01000022.1 | 100 | 3298 | 0 | 0 | 1 | 3298 | 440663 | 443960 | 0 | 6091 | Ec12-0466 |
| AIPQ01000028.1 | 100 | 3298 | 0 | 0 | 1 | 3298 | 154036 | 150739 | 0 | 6091 | Ec12-0465 |
| AHPA01000012.1 | 100 | 3298 | 0 | 0 | 1 | 3298 | 80649 | 83946 | 0 | 6091 |  |
| AHOZ01000018.1 | 100 | 3298 | 0 | 0 | 1 | 3298 | 80745 | 84042 | 0 | 6091 |  |
| AHOY01000021.1 | 100 | 3298 | 0 | 0 | 1 | 3298 | 440553 | 443850 | 0 | 6091 |  |
| AHOX01000013.1 | 100 | 3298 | 0 | 0 | 1 | 3298 | 80745 | 84042 | 0 | 6091 |  |
| AHOW01000021.1 | 100 | 3298 | 0 | 0 | 1 | 3298 | 80860 | 84157 | 0 | 6091 |  |
| AHOV01000018.1 | 100 | 3298 | 0 | 0 | 1 | 3298 | 152370 | 155667 | 0 | 6091 |  |
| AHOU01000017.1 | 100 | 3298 | 0 | 0 | 1 | 3298 | 80909 | 84206 | 0 | 6091 |  |
| AGWH01000013.1 | 100 | 3298 | 0 | 0 | 1 | 3298 | 81285 | 84582 | 0 | 6091 | Ec11-9941 |
| AGWG01000027.1 | 100 | 3298 | 0 | 0 | 1 | 3298 | 80745 | 84042 | 0 | 6091 | Ec11-9990 |
| AGWF01000028.1 | 100 | 3298 | 0 | 0 | 1 | 3298 | 153775 | 150478 | 0 | 6091 | Ec11-9459 |
| AFWC01000250.1 | 100 | 3273 | 0 | 0 | 1 | 3298 | 81101 | 84398 | 0 | 6091 |  |
| AFWB01000471.1 | 100 | 3272 | 0 | 0 | 1 | 3298 | 3957 | 7254 | 0 | 6091 |  |
| AFVR01000082.1 | 100 | 3272 | 0 | 0 | 1 | 3298 | 4139 | 7436 | 0 | 6091 | TY2482 |
| AFST01000007.2 | 100 | 3272 | 0 | 0 | 1 | 3298 | 155552 | 158849 | 0 | 6091 |  |
| AFSO01000030.1 | 100 | 3272 | 0 | 0 | 1 | 3298 | 81970 | 85267 | 0 | 6091 |  |
| AFPN02000020.1 | 100 | 3272 | 0 | 0 | 1 | 3298 | 150906 | 154203 | 0 | 6091 |  |
| AFOB02000300.1 | 100 | 3272 | 0 | 0 | 1 | 3298 | 4645 | 1348 | 0 | 6091 |  |
| NC_019091.1 | 100 | 3298 | 0 | 0 | 1 | 3298 | 5048 | 1751 | 0 | 6091 | pASL01a |
| NC_018650.1 | 100 | 3298 | 0 | 0 | 1 | 3298 | 85954 | 89251 | 0 | 6091 | 2009EL-2050 |
| NC_018658.1 | 100 | 3298 | 0 | 0 | 1 | 3298 | 87387 | 84090 | 0 | 6091 | 2011C-3493 |
| NC_018661.1 | 100 | 3298 | 0 | 0 | 1 | 3298 | 76383 | 79680 | 0 | 6091 | 2009EL-2071 |
| AMWA01000006.1 | 100 | 3298 | 0 | 0 | 1 | 3298 | 150611 | 153908 | 0 | 6091 |  |
| AMVZ01000009.1 | 100 | 3298 | 0 | 0 | 1 | 3298 | 4363 | 7660 | 0 | 6091 |  |
| AMVY01000007.1 | 100 | 3298 | 0 | 0 | 1 | 3298 | 84267 | 87564 | 0 | 6091 |  |
