# Supplementary material for: A Role for Tn6029 in the Evolution of the Complex Antibiotic Resistance Gene Loci in Genomic Island 3 in Enteroaggregative Hemorrhagic Escherichia coli O104:H4
Source: PLoS One. 2015 Feb 12;10(2):e0115781. doi: 10.1371/journal.pone.0115781 (PMC4326458; doi:10.1371/journal.pone.0115781)
Supplement: S3 Table — (DOCX) [file pone.0115781.s005.docx]

**Table 3: Results of BLASTn analysis using Fragment 3 (8677nt)**

| **Subject ID** | **%**  **identity** | **Alignment length** | **Mismatches** | **Gaps in align** | **Query start** | **Query**  **end** | **Subject Start** | **Subject End** | **E-Value** | **Bit**  **Score** | **Genomes** |
| --- | --- | --- | --- | --- | --- | --- | --- | --- | --- | --- | --- |
|  |  |  |  |  |  |  |  |  |  |  |  |
| AHOY01000021.1 | 100 | 8677 | 0 | 0 | 1 | 8677 | 443731 | 452407 | 0 | 16024 |  |
| AHOW01000021.1 | 100 | 8677 | 0 | 0 | 1 | 8677 | 84038 | 92714 | 0 | 16024 |  |
| NC_018650.1 | 100 | 8677 | 0 | 0 | 1 | 8677 | 85458 | 76782 | 0 | 16024 | 2009EL-2050 |
| AHOU01000017.1 | 100 | 7952 | 0 | 0 | 1 | 7952 | 84087 | 92038 | 0 | 14685 |  |
| NC_013365.1 | 100 | 7857 | 0 | 0 | 821 | 8677 | 114896 | 107040 | 0 | 14510 |  |
| NC_013728.1 | 99.99 | 7857 | 1 | 0 | 821 | 8677 | 7268 | 15124 | 0 | 14504 |  |
| NC_022996.1 | 99.97 | 7857 | 1 | 1 | 821 | 8677 | 12351 | 20206 | 0 | 14497 |  |
| NC_022992.1 | 99.97 | 7857 | 1 | 1 | 821 | 8677 | 12351 | 20206 | 0 | 14497 |  |
| AFVA01000010.1 | 100 | 7243 | 0 | 0 | 821 | 8063 | 1 | 7243 | 0 | 13376 |  |
| AFRM01000016.1 | 99.95 | 6510 | 1 | 2 | 2170 | 8677 | 1 | 6510 | 0 | 12004 |  |
| AMWA01000007.1 | 100 | 6508 | 0 | 0 | 2170 | 8677 | 1 | 6508 | 0 | 12019 |  |
| AMVZ01000010.1 | 100 | 6508 | 0 | 0 | 2170 | 8677 | 1 | 6508 | 0 | 12019 |  |
| AMVY01000008.1 | 100 | 6508 | 0 | 0 | 2170 | 8677 | 1 | 6508 | 0 | 12019 |  |
| AMVX01000005.1 | 100 | 6508 | 0 | 0 | 2170 | 8677 | 1 | 6508 | 0 | 12019 |  |
| AMVW01000017.1 | 100 | 6508 | 0 | 0 | 2170 | 8677 | 1 | 6508 | 0 | 12019 |  |
| AMVV01000011.1 | 100 | 6508 | 0 | 0 | 2170 | 8677 | 1 | 6508 | 0 | 12019 |  |
| AMVU01000004.1 | 100 | 6508 | 0 | 0 | 2170 | 8677 | 1 | 6508 | 0 | 12019 |  |
| AMVT01000003.1 | 100 | 6508 | 0 | 0 | 2170 | 8677 | 1 | 6508 | 0 | 12019 |  |
| AMVS01000014.1 | 100 | 6508 | 0 | 0 | 2170 | 8677 | 1 | 6508 | 0 | 12019 |  |
| AMVR01000010.1 | 100 | 6508 | 0 | 0 | 2170 | 8677 | 1 | 6508 | 0 | 12019 |  |
| AIPR01000023.1 | 100 | 6508 | 0 | 0 | 2170 | 8677 | 1 | 6508 | 0 | 12019 | Ec12-0466 |
| AHPA01000013.1 | 100 | 6508 | 0 | 0 | 2170 | 8677 | 1 | 6508 | 0 | 12019 |  |
| AFRH01000012.1 | 100 | 6508 | 0 | 0 | 2170 | 8677 | 1 | 6508 | 0 | 12019 |  |
| AFUY01000021.1 | 99.98 | 6508 | 1 | 0 | 2170 | 8677 | 1 | 6508 | 0 | 12013 |  |
| AIPQ01000028.1 | 100 | 6030 | 0 | 0 | 2648 | 8677 | 157179 | 163208 | 0 | 11136 | Ec12-0465 |
| AHOV01000018.1 | 100 | 6030 | 0 | 0 | 2648 | 8677 | 158195 | 164224 | 0 | 11136 |  |
| NC_019091.1 | 100 | 6030 | 0 | 0 | 2648 | 8677 | 26306 | 20277 | 0 | 11136 |  |
| NC_018658.1 | 100 | 6030 | 0 | 0 | 2648 | 8677 | 83395 | 77366 | 0 | 11136 | 20011C-3493 |
| NC_018661.1 | 100 | 6030 | 0 | 0 | 2648 | 8677 | 73240 | 67211 | 0 | 11136 |  |
| AFRI01000011.1 | 99.98 | 6027 | 0 | 1 | 2652 | 8677 | 1 | 6027 | 0 | 11123 |  |
| AFUZ01000022.1 | 100 | 5995 | 0 | 0 | 2683 | 8677 | 1 | 5995 | 0 | 11071 |  |
| AHOX01000013.1 | 100 | 5407 | 0 | 0 | 2648 | 8054 | 86570 | 91976 | 0 | 9985 |  |
| AHOZ01000019.1 | 100 | 5301 | 0 | 0 | 3377 | 8677 | 1 | 5301 | 0 | 9790 |  |
| AGWF01000030.1 | 100 | 5301 | 0 | 0 | 3377 | 8677 | 1 | 5301 | 0 | 9790 | Ec11-9459 |
| AFVE01000010.1 | 100 | 5301 | 0 | 0 | 3377 | 8677 | 1 | 5301 | 0 | 9790 |  |
| AFVD01000033.1 | 100 | 5301 | 0 | 0 | 3377 | 8677 | 1 | 5301 | 0 | 9790 |  |
| AFVB01000007.1 | 100 | 5301 | 0 | 0 | 3377 | 8677 | 1 | 5301 | 0 | 9790 |  |
| AFUX01000023.1 | 100 | 5301 | 0 | 0 | 3377 | 8677 | 1 | 5301 | 0 | 9790 |  |
| AIPQ01000028.1 | 100 | 3471 | 0 | 0 | 1 | 3471 | 158002 | 154532 | 0 | 6410 |  |
| AHOV01000018.1 | 100 | 3471 | 0 | 0 | 1 | 3471 | 159018 | 155548 | 0 | 6410 |  |
| NC_018661.1 | 100 | 3471 | 0 | 0 | 1 | 3471 | 72417 | 75887 | 0 | 6410 | 2009EL-2071 |
