# Supplementary material for: A Role for Tn6029 in the Evolution of the Complex Antibiotic Resistance Gene Loci in Genomic Island 3 in Enteroaggregative Hemorrhagic Escherichia coli O104:H4
Source: PLoS One. 2015 Feb 12;10(2):e0115781. doi: 10.1371/journal.pone.0115781 (PMC4326458; doi:10.1371/journal.pone.0115781)
Supplement: S4 Table — (DOCX) [file pone.0115781.s006.docx]

**Table 4: Results of BLASTn analysis using Fragment 4 (4670nt)**

| **Subject ID** | **%**  **identity** | **Alignment length** | **Mismatches** | **Gaps in align** | **Querry start** | **Querry end** | **Subject Start** | **Subject End** | **E-Value** | **Bit Score** | **Genomes** |
| --- | --- | --- | --- | --- | --- | --- | --- | --- | --- | --- | --- |
|  |  |  |  |  |  |  |  |  |  |  |  |
| AMWA01000007.1 | 100 | 4670 | 0 | 0 | 1 | 4670 | 12504 | 7835 | 0 | 8624 |  |
| AMVZ01000010.1 | 100 | 4670 | 0 | 0 | 1 | 4670 | 12504 | 7835 | 0 | 8624 |  |
| AMVY01000008.1 | 100 | 4670 | 0 | 0 | 1 | 4670 | 12504 | 7835 | 0 | 8624 |  |
| AMVX01000005.1 | 100 | 4670 | 0 | 0 | 1 | 4670 | 12504 | 7835 | 0 | 8624 |  |
| AMVW01000017.1 | 100 | 4670 | 0 | 0 | 1 | 4670 | 12504 | 7835 | 0 | 8624 |  |
| AMVV01000011.1 | 100 | 4670 | 0 | 0 | 1 | 4670 | 12504 | 7835 | 0 | 8624 |  |
| AMVT01000003.1 | 100 | 4670 | 0 | 0 | 1 | 4670 | 12504 | 7835 | 0 | 8624 |  |
| AMVS01000014.1 | 100 | 4670 | 0 | 0 | 1 | 4670 | 12504 | 7835 | 0 | 8624 |  |
| AMVR01000010.1 | 100 | 4670 | 0 | 0 | 1 | 4670 | 12504 | 7835 | 0 | 8624 |  |
| AIPR01000023.1 | 100 | 4670 | 0 | 0 | 1 | 4670 | 12504 | 7835 | 0 | 8624 | Ec12-0466 |
| AIPQ01000028.1 | 100 | 4670 | 0 | 0 | 1 | 4670 | 169204 | 164535 | 0 | 8624 | Ec12-0465 |
| AHPA01000013.1 | 100 | 4670 | 0 | 0 | 1 | 4670 | 12504 | 7835 | 0 | 8624 |  |
| AHOZ01000019.1 | 100 | 4670 | 0 | 0 | 1 | 4670 | 11297 | 6628 | 0 | 8624 |  |
| AHOY01000021.1 | 100 | 4670 | 0 | 0 | 1 | 4670 | 458403 | 453734 | 0 | 8624 |  |
| AHOW01000021.1 | 100 | 4670 | 0 | 0 | 1 | 4670 | 98710 | 94041 | 0 | 8624 |  |
| AHOV01000018.1 | 100 | 4670 | 0 | 0 | 1 | 4670 | 170220 | 165551 | 0 | 8624 |  |
| AGWF01000030.1 | 100 | 4670 | 0 | 0 | 1 | 4670 | 11297 | 6628 | 0 | 8624 | Ec11-9459 |
| AFVR01000014.1 | 100 | 4670 | 0 | 0 | 1 | 4670 | 5045 | 376 | 0 | 8624 | TY2482 |
| AFVE01000011.1 | 100 | 4670 | 0 | 0 | 1 | 4670 | 5428 | 759 | 0 | 8624 |  |
| AFVD01000033.1 | 100 | 4670 | 0 | 0 | 1 | 4670 | 11297 | 6628 | 0 | 8624 |  |
| AFVC01000023.1 | 100 | 4670 | 0 | 0 | 1 | 4670 | 6816 | 2147 | 0 | 8624 |  |
| AFVB01000007.1 | 100 | 4670 | 0 | 0 | 1 | 4670 | 11297 | 6628 | 0 | 8624 |  |
| AFUY01000021.1 | 100 | 4670 | 0 | 0 | 1 | 4670 | 12504 | 7835 | 0 | 8624 |  |
| AFUX01000023.1 | 100 | 4670 | 0 | 0 | 1 | 4670 | 11297 | 6628 | 0 | 8624 |  |
| AFRM01000016.1 | 100 | 4670 | 0 | 0 | 1 | 4670 | 12506 | 7837 | 0 | 8624 |  |
| AFRI01000011.1 | 100 | 4670 | 0 | 0 | 1 | 4670 | 12023 | 7354 | 0 | 8624 |  |
| AFRH01000012.1 | 100 | 4670 | 0 | 0 | 1 | 4670 | 12504 | 7835 | 0 | 8624 |  |
| AFOB02000328.1 | 100 | 4670 | 0 | 0 | 1 | 4670 | 1635 | 6304 | 0 | 8624 |  |
| NC_018650.1 | 100 | 4670 | 0 | 0 | 1 | 4670 | 70792 | 75461 | 0 | 8624 | 2009EL-2050 |
| NC_018658.1 | 100 | 4670 | 0 | 0 | 1 | 4670 | 71370 | 76039 | 0 | 8624 | 20011C-3493 |
| AFST01000007.2 | 99.98 | 4670 | 0 | 1 | 1 | 4670 | 173397 | 168729 | 0 | 8617 |  |
| AFVS01000784.1 | 99.89 | 4667 | 1 | 4 | 1 | 4664 | 288 | 4953 | 0 | 8589 |  |
| AHOX01000014.1 | 100 | 4658 | 0 | 0 | 1 | 4658 | 4658 | 1 | 0 | 8602 |  |
| AHOU01000019.1 | 100 | 4658 | 0 | 0 | 1 | 4658 | 4658 | 1 | 0 | 8602 |  |
| AFVA01000012.1 | 100 | 4658 | 0 | 0 | 1 | 4658 | 4658 | 1 | 0 | 8602 |  |
| AFUZ01000023.1 | 100 | 4658 | 0 | 0 | 1 | 4658 | 4658 | 1 | 0 | 8602 |  |
| AMWA01000007.1 | 100 | 4670 | 0 | 0 | 1 | 4670 | 12504 | 7835 | 0 | 8624 |  |
