# Supplementary material for: A Role for Tn6029 in the Evolution of the Complex Antibiotic Resistance Gene Loci in Genomic Island 3 in Enteroaggregative Hemorrhagic Escherichia coli O104:H4
Source: PLoS One. 2015 Feb 12;10(2):e0115781. doi: 10.1371/journal.pone.0115781 (PMC4326458; doi:10.1371/journal.pone.0115781)
Supplement: S5 Table — (DOCX) [file pone.0115781.s007.docx]

**Table 5: Results of BLASTn analysis using Fragment 5 (5301nt)**

| **Subject ID** | **%**  **identity** | **Alignment length** | **Mismatches** | **Gaps in align** | **Querry start** | **Querry end** | **Subject Start** | **Subject End** | **E-Value** | **Bit Score** | **Genomes** |
| --- | --- | --- | --- | --- | --- | --- | --- | --- | --- | --- | --- |
|  |  |  |  |  |  |  |  |  |  |  |  |
| AIPR01000023.1 | 100 | 5301 | 0 | 0 | 1 | 5301 | 12152 | 17452 | 0 | 9790 |  |
| AIPQ01000028.1 | 100 | 5301 | 0 | 0 | 1 | 5301 | 168852 | 174152 | 0 | 9790 |  |
| AGWF01000030.1 | 100 | 5301 | 0 | 0 | 1 | 5301 | 10945 | 16245 | 0 | 9790 |  |
| NC_018650.1 | 100 | 5301 | 0 | 0 | 1 | 5301 | 71144 | 65844 | 0 | 9790 |  |
| AFRI01000011.1 | 99.64 | 4783 | 0 | 14 | 536 | 5301 | 13555 | 18337 | 0 | 8722 |  |
| AFWC01000253.1 | 99.64 | 4782 | 0 | 12 | 536 | 5301 | 650 | 5430 | 0 | 8720 |  |
| AFPN02000022.1 | 99.83 | 4774 | 0 | 3 | 536 | 5301 | 6097 | 10870 | 0 | 8765 |  |
| AMWA01000007.1 | 99.87 | 4772 | 0 | 1 | 536 | 5301 | 14036 | 18807 | 0 | 8774 |  |
| AMVZ01000010.1 | 99.87 | 4772 | 0 | 1 | 536 | 5301 | 14036 | 18807 | 0 | 8774 |  |
| AMVY01000008.1 | 99.87 | 4772 | 0 | 1 | 536 | 5301 | 14036 | 18807 | 0 | 8774 |  |
| AMVX01000005.1 | 99.87 | 4772 | 0 | 1 | 536 | 5301 | 14036 | 18807 | 0 | 8774 |  |
| AMVW01000017.1 | 99.87 | 4772 | 0 | 1 | 536 | 5301 | 14036 | 18807 | 0 | 8774 |  |
| AMVV01000011.1 | 99.87 | 4772 | 0 | 1 | 536 | 5301 | 14036 | 18807 | 0 | 8774 |  |
| AMVT01000003.1 | 99.87 | 4772 | 0 | 1 | 536 | 5301 | 14036 | 18807 | 0 | 8774 |  |
| AMVS01000014.1 | 99.87 | 4772 | 0 | 1 | 536 | 5301 | 14036 | 18807 | 0 | 8774 |  |
| AMVR01000010.1 | 99.87 | 4772 | 0 | 1 | 536 | 5301 | 14036 | 18807 | 0 | 8774 |  |
| AHPA01000013.1 | 99.87 | 4772 | 0 | 1 | 536 | 5301 | 14036 | 18807 | 0 | 8774 |  |
| AHOZ01000019.1 | 99.87 | 4772 | 0 | 1 | 536 | 5301 | 12829 | 17600 | 0 | 8774 |  |
| AHOY01000021.1 | 99.87 | 4772 | 0 | 1 | 536 | 5301 | 459935 | 464706 | 0 | 8774 |  |
| AHOX01000014.1 | 99.87 | 4772 | 0 | 1 | 536 | 5301 | 6190 | 10961 | 0 | 8774 |  |
| AHOW01000021.1 | 99.87 | 4772 | 0 | 1 | 536 | 5301 | 100242 | 105013 | 0 | 8774 |  |
| AHOV01000018.1 | 99.87 | 4772 | 0 | 1 | 536 | 5301 | 171752 | 176523 | 0 | 8774 |  |
| AHOU01000019.1 | 99.87 | 4772 | 0 | 1 | 536 | 5301 | 6190 | 10961 | 0 | 8774 |  |
| AFVE01000011.1 | 99.87 | 4772 | 0 | 1 | 536 | 5301 | 6960 | 11731 | 0 | 8774 |  |
| AFVC01000023.1 | 99.87 | 4772 | 0 | 1 | 536 | 5301 | 8348 | 13119 | 0 | 8774 |  |
| AFVB01000007.1 | 99.87 | 4772 | 0 | 1 | 536 | 5301 | 12829 | 17600 | 0 | 8774 |  |
| AFVA01000012.1 | 99.87 | 4772 | 0 | 1 | 536 | 5301 | 6190 | 10961 | 0 | 8774 |  |
| AFUX01000023.1 | 99.87 | 4772 | 0 | 1 | 536 | 5301 | 12829 | 17600 | 0 | 8774 |  |
| AFSO01000032.1 | 99.87 | 4772 | 0 | 1 | 536 | 5301 | 6095 | 10866 | 0 | 8774 |  |
| AFRM01000016.1 | 99.87 | 4772 | 0 | 1 | 536 | 5301 | 14038 | 18809 | 0 | 8774 |  |
| AFRH01000012.1 | 99.87 | 4772 | 0 | 1 | 536 | 5301 | 14036 | 18807 | 0 | 8774 |  |
| AFOG01000085.1 | 99.87 | 4772 | 0 | 1 | 536 | 5301 | 49 | 4820 | 0 | 8774 |  |
| NC_018658.1 | 99.87 | 4772 | 0 | 1 | 536 | 5301 | 69838 | 65067 | 0 | 8774 |  |
| AFST01000007.2 | 99.81 | 4771 | 0 | 4 | 537 | 5301 | 174923 | 179690 | 0 | 8752 |  |
| AFRO01000331.1 | 99.87 | 4761 | 0 | 1 | 547 | 5301 | 4801 | 41 | 0 | 8754 |  |
| AIPR01000023.1 | 100 | 5301 | 0 | 0 | 1 | 5301 | 12152 | 17452 | 0 | 9790 |  |
| AIPQ01000028.1 | 100 | 5301 | 0 | 0 | 1 | 5301 | 168852 | 174152 | 0 | 9790 |  |
